# Third-Generation Tetracyclines: Current Knowledge and Therapeutic Potential

**DOI:** 10.3390/biom14070783

**Published:** 2024-06-30

**Authors:** Dimitris Kounatidis, Maria Dalamaga, Eugenia Grivakou, Irene Karampela, Petros Koufopoulos, Vasileios Dalopoulos, Nikolaos Adamidis, Eleni Mylona, Aikaterini Kaziani, Natalia G. Vallianou

**Affiliations:** 1 Diabetes Center, First Department of Propaedeutic Internal Medicine, Laiko General Hospital, Medical School, National and Kapodistrian University of Athens, 11527 Athens, Greece; dimitriskounatidis82@outlook.com; 2 Department of Biological Chemistry, Medical School, National and Kapodistrian University of Athens, 11527 Athens, Greece; madalamaga@med.uoa.gr; 3 Department of Internal Medicine, Evangelismos General Hospital, 10676 Athens, Greece; eugeniagriv@yahoo.gr (E.G.); mylonaelena@gmail.com (E.M.); katkaziani@hotmail.com (A.K.); 4 Second Department of Critical Care, Attikon General University Hospital, Medical School, National and Kapodistrian University of Athens, 12462 Athens, Greece; eikaras1@gmail.com; 5 First Department of Internal Medicine, Sismanogleio General Hospital, 15126 Athens, Greece; peterkouf13@gmail.com (P.K.); billydalo@hotmail.gr (V.D.); nikos.adamidis7@gmail.com (N.A.)

**Keywords:** tetracyclines, tigecycline, eravacycline, omadacycline, mechanism of action, resistance, non-antibiotic properties, malignancy, immunomodulation

## Abstract

Tetracyclines constitute a unique class of antibiotic agents, widely prescribed for both community and hospital infections due to their broad spectrum of activity. Acting by disrupting protein synthesis through tight binding to the 30S ribosomal subunit, their interference is typically reversible, rendering them bacteriostatic in action. Resistance to tetracyclines has primarily been associated with changes in pump efflux or ribosomal protection mechanisms. To address this challenge, tetracycline molecules have been chemically modified, resulting in the development of third-generation tetracyclines. These novel tetracyclines offer significant advantages in treating infections, whether used alone or in combination therapies, especially in hospital settings. Beyond their conventional antimicrobial properties, research has highlighted their potential non-antibiotic properties, including their impact on immunomodulation and malignancy. This review will focus on third-generation tetracyclines, namely tigecycline, eravacycline, and omadacycline. We will delve into their mechanisms of action and resistance, while also evaluating their pros and cons over time. Additionally, we will explore their therapeutic potential, analyzing their primary indications of prescription, potential future uses, and non-antibiotic features. This review aims to provide valuable insights into the clinical applications of third-generation tetracyclines, thereby enhancing understanding and guiding optimal clinical use.

## 1. Introduction 

Since the early 1940s, aureomycin was the first compound in the tetracycline family discovered by physician Benjamin Minge Duggar. Due to its yellow color, it was named aureomycin and received Food and Drug Administration (FDA) approval as a broad-spectrum antibiotic in 1948 [1]. The second antibiotic in this group was terramycin, which gained FDA approval in 1950 and was slightly more water-soluble than aureomycin. However, both compounds, being natural substances, had disadvantages regarding their bioavailability [2]. To address these limitations, Lloyd Conover developed the first chemical compound based on aureomycin’s structure, aiming to improve its pharmacokinetic profile and potency. This hemisynthetic compound was approved by the FDA for clinical use in 1954 under the name tetracycline [3].

Since then, tetracycline has been widely used successfully for various diseases. Another first-generation analog, demeclocycline, has been developed and is considered a therapeutic option for cases of Syndrome of Inappropriate Antidiuretic Hormone Secretion (SIADH) [4]. Subsequently, doxycycline was approved by the FDA in 1967 and has become the most widely used tetracycline [5]. Minocycline is also notable, primarily used for treating acne vulgaris and sexually transmitted diseases. Recently, the FDA has approved its use as part of combination therapy for infections caused by multidrug-resistant (MDR) *Acinetobacter* species. Minocycline has been extensively studied for its pleiotropic non-antibiotic properties, including antioxidant and anti-apoptotic effects, as well as its role in regulating immune cell activation and proliferation [6,7,8].

Due to high resistance rates attributed mainly to efflux pumps, a new category of glycylcyclines was discovered. Tigecycline (TIG) received FDA approval in 2005, followed by eravacycline (ERV) in August 2018 [9]. Omadacycline (OMC), the first drug in the aminomethylcycline category, was approved in October 2018 for the treatment of community-acquired bacterial pneumonia (CABP) and acute bacterial skin and soft tissue infections (ABSSSIs) [9]. Collectively, tigecycline, eravacycline, and omadacycline represent third-generation tetracyclines. On the other hand, sarecycline, the fourth member of this generation, has a narrow spectrum of activity and is exclusively used for treating acne vulgaris. Therefore, it will not be discussed further in this review article [10]. Table 1 categorizes the tetracycline family into three generations and lists the main representative members of each generation.

Tetracyclines constitute a distinctive class of antibiotics with applications spanning a wide spectrum of infections caused by both Gram-positive and Gram-negative bacteria. Moreover, they have demonstrated effectiveness against intracellular organisms and protozoan parasites [12]. Functionally, tetracyclines inhibit bacterial growth by impeding protein biosynthesis [13]. While they are typically considered bacteriostatic antibiotics at therapeutic concentrations due to their reversible interaction with ribosomes, recent studies have suggested bactericidal effects in vitro, especially with third-generation tetracyclines [9,14].

The aim of this review is to shed light on the pharmacological and clinical characteristics of third-generation tetracyclines, encompassing their mechanisms of action, their resistance mechanisms, and their evolution over time. Additionally, we will explore their main indications of prescription and their therapeutic potential, including their promising role in immunomodulation and malignancy.

## 2. Literature Search Methodology 

For the preparation of this narrative review, we conducted an extensive search in the PubMed NIH database using the search terms “tetracyclines”, “tigecycline”, “eravacycline”, and “omadacycline”. Our search was confined to items published within the past 10 years. We primarily concentrated on research and review articles, randomized clinical trials, and meta-analyses. Furthermore, we scrutinized the references of these articles to uncover other pertinent publications. Given the substantial number of manuscripts retrieved, it is acknowledged that not all of them can be comprehensively covered within the scope of this review.

## 3. Chemical Structure

**Figure 1:**
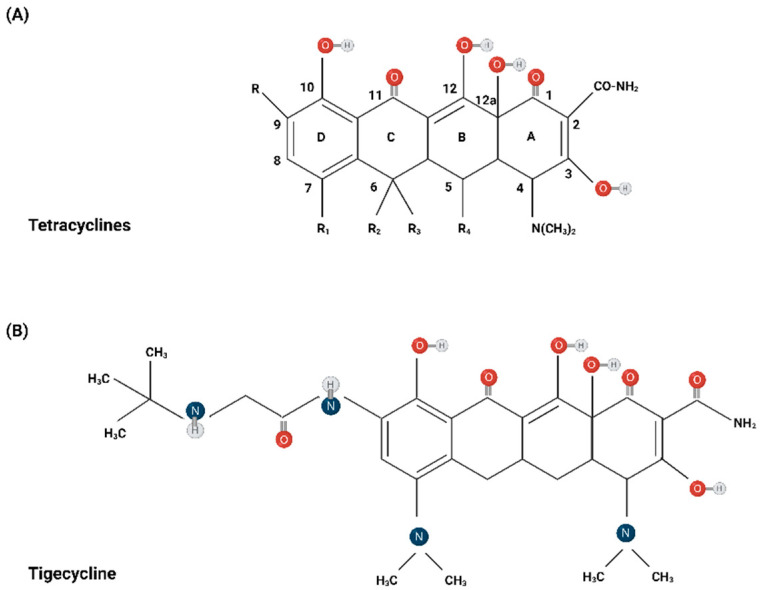

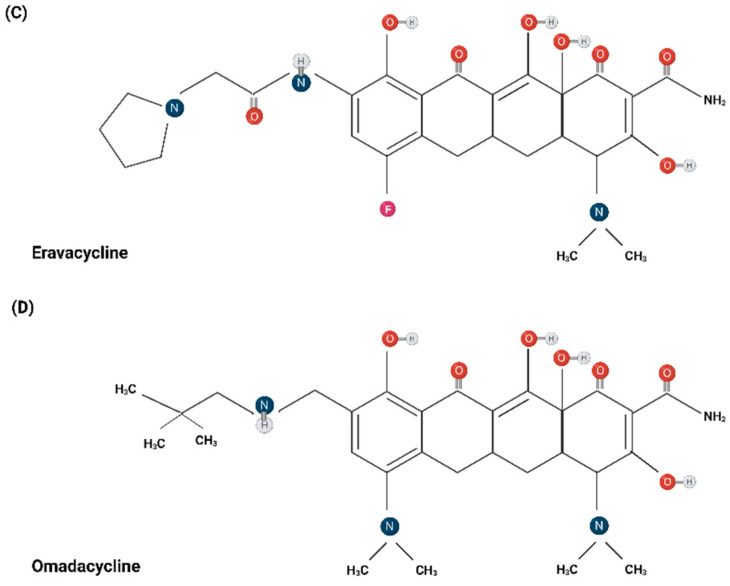
The chemical structure of tetracyclines (**A**), including their third-generation analogs, namely tigecycline (**B**), eravacycline (**C**), and omadacycline (**D**) [12,13,14,15,16,17]. Created with BioRender.com (accessed on 26 June 2024).

The fundamental structure of tetracyclines comprises four benzene rings linearly condensed within a hydronaphthacene nucleus. Variations among analogs within this class primarily stem from differences in substituents at positions C5, C6, C7, and C9 [12]. Modifications in this chemical structure have led to the development of novel third-generation tetracyclines. Tigecycline, a synthetic derivative of minocycline, features a glycylamido moiety attached to the 9-position of the tetracycline ring, a substitution pattern absent in any naturally occurring or semi-synthetic tetracycline. This alteration renders the compound effective against the two major tetracycline resistance mechanisms: ribosomal protection proteins and efflux pumps [15]. Eravacycline, a synthetic fluorocycline, shares structural similarities with tigecycline but features two modifications to the D-ring of its tetracycline core. Specifically, it replaces the dimethylamine group at C-7 with a fluorine atom and substitutes the 2-tertiary-butyl glycylamido at C-9 with a pyrrolidinoacetamido group. On the other hand, omadacycline is an aminomethylcycline which serves as a modified minocycline molecule with chemical substitution at the C-9 position of the D-ring of the minocycline core [16]. Unlike tigecycline and eravacycline, which possess glycylamido substitution at this site, omadacycline incorporates an aminomethyl group. This modification enhances the molecule’s bioavailability [17]. Figure 1 illustrates the standard chemical structure and traditional numbering of the condensed rings and important positions of tetracyclines, alongside the chemical structure of third-generation tetracyclines. 

## 4. Mechanism of Action 

Tetracyclines exert their antibacterial effects by disrupting protein synthesis. They tightly bind to the 16S rRNA site on the 30S ribosomal subunit during translation, preventing the binding of aminoacyl-tRNA to the bacterial ribosome. This interference inhibits the entry of aminoacyl-tRNA into the acceptor site (A) on the ribosome, disrupting the incorporation of amino acid residues during polypeptide chain formation. Consequently, bacterial protein synthesis is halted, leading to the inhibition of bacterial growth [13]. The mechanism of action of tetracyclines is generally bacteriostatic because the interaction between tetracyclines and ribosomes is reversible [18]. However, in vitro evidence has shown that third-generation tetracyclines may exert bactericidal activity [14]. 

In Gram-positive bacteria, which typically have a single lipid bilayer cell membrane, tetracyclines can penetrate the cytoplasm through both passive diffusion and active transport mechanisms. Once inside the cytoplasm, tetracyclines bind with Mg^2+^ ions, facilitating effective ribosomal targeting [12]. On the other hand, Gram-negative bacteria possess an additional outer membrane composed of lipopolysaccharides (LPS), serving as a barrier to tetracycline entry. To overcome this barrier, tetracyclines primarily penetrate the cell through outer membrane porins (OMPs), such as OmpF and OmpC. It is believed that tetracyclines traverse these porin channels as magnesium–tetracycline coordination complexes [19,20]. Subsequently, free tetracycline dissociates and diffuses through the lipid bilayer of the cytoplasmic membrane. Alternatively, tetracyclines may enter bacterial cells via passive diffusion or active transport mechanisms, with the latter requiring both ATP and Mg^2+^ for active uptake [19,20].

Tigecycline and eravacycline inhibit bacterial growth by targeting the ribosomal 30S subunit, hindering the entry of aminoacyl-tRNA molecules into the A-site and preventing the incorporation of amino acids into peptide chains. Tigecycline’s potency surpasses that of minocycline and traditional tetracyclines by three to twenty times [21,22]. Eravacycline, similarly, binds to the ribosomal 30S subunit, disrupting protein synthesis and demonstrating a tenfold higher affinity for ribosomal binding compared to tetracycline [23]. It exhibits in vitro bactericidal activity against strains of *Acinetobacter baumannii*, *Escherichia coli*, and *Klebsiella pneumoniae* [16]. In biophysical experiments utilizing purified ribosomes, it was observed that omadacycline exhibits a binding affinity to 70S ribosomes similar to minocycline [24]. Omadacycline, akin to tigecycline, remains effective against tetracycline resistance mechanisms like efflux and ribosomal protection, retaining activity in the presence of the ribosomal protection protein Tet(O) [24]. 

## 5. Clinical Pharmacology 

### 5.1. Tigecycline 

Tigecycline’s clinical pharmacology indicates exclusive intravenous administration due to poor oral absorption. It exhibits high plasma protein binding (71–89%), a lengthy half-life (55.8 h), and extensive distribution into tissues, including the lungs, cerebrospinal fluid, liver, and kidneys. Primarily excreted unchanged in the gall bladder, tigecycline undergoes minimal liver metabolism. Dosage adjustments are unnecessary for patients with compensated or moderately decompensated cirrhosis. Tigecycline’s pharmacokinetics are unaffected by various factors such as age, gender, race, renal disease, and food intake. It is mainly eliminated through feces (59%) and urine (32%). Clinical trials show good tolerance for up to 11.5 days, with common adverse reactions including nausea, vomiting, and diarrhea. Given its structural similarity to tetracyclines, precautions regarding potential adverse effects of the tetracycline class are included in its label [25,26,27]. Tigecycline is commonly administered in an initial loading dose of 100 mg, followed by a maintenance dose of 50 mg given twice daily [28]. However, pharmacokinetic studies have revealed that standard blood levels of tigecycline may not suffice for treating *Acinetobacter baumannii* bacteremia. Hence, clinical practice often involves administering higher doses, with double doses recommended. Specifically, high-dose tigecycline regimens, such as a loading dose of 200 mg followed by 100 mg twice daily, have been suggested. Research has indicated that these higher doses are more effective compared to standard tigecycline dosing [29].

### 5.2. Eravacycline

Eravacycline’s pharmacokinetics have been extensively studied in both Phase I and Phase II trials, whether administered intravenously or orally. Oral bioavailability hovers around 28%, with an elimination half-life ranging from 22 to 34 h and a steady-state volume of distribution of about 3.3–4.2 L/kg. Protein binding ranges from 79% to 90%, with renal excretion contributing to 16% of total clearance. Intravenous eravacycline displays linear pharmacokinetics, modeled by a four-compartment model. Population studies of intravenous administration indicate a mean steady-state volume of distribution of 4.2 L/kg, a mean terminal elimination half-life of 48 h, and a mean total clearance of 13.5 L/h [16,30].

Notably, eravacycline’s antibacterial activity has been assessed in vitro on *Acinetobacter baumannii*, *Escherichia coli*, and *Klebsiella pneumoniae*, exhibiting bacteriostatic effects at concentrations 2–8 times the MIC and bactericidal effects at concentrations ranging from 1–16 mg/L for *Acinetobacter baumannii*, 0.25–2 mg/L for *Escherichia coli*, and 0.5–8 mg/L for *Klebsiella pneumoniae*, across various resistance genes and phenotypes [31]. Eravacycline is typically administered intravenously at a dosage of 1 mg/kg every 12 h. The duration of treatment typically ranges from 4 to 14 days, depending on the severity of the infection being treated and the patient’s response to therapy [32].

### 5.3. Omadacycline 

Omadacycline is available in both oral and intravenous forms. Its oral bioavailability is relatively low at 34.5%, further decreasing when taken with food or calcium. Loading doses are necessary to achieve therapeutic levels by the second day of treatment, given its elimination half-life of 13 to 16 h. Unlike other tetracyclines with high plasma protein binding, omadacycline binds weakly to human plasma proteins at 21%. It distributes extensively in various tissues, including the lungs, liver, and kidneys, with concentrations exceeding those in the bloodstream after oral or intravenous administration [33,34]. Metabolically, omadacycline does not undergo cytochrome P450 enzyme metabolism or interact with drug transporters, resulting in a low risk of drug interactions. However, at higher concentrations, it may moderately inhibit the OAT1 transporter, with potential inhibition reaching up to 32% [33]. Primary excretion occurs via feces, with no need for dose adjustment in hepatic or renal impairment. Gender is the only demographic significantly affecting drug pharmacokinetics, with systemic clearance 16% lower in females than males. Comorbidities, including cardiovascular disease, do not notably impact omadacycline pharmacokinetics, except for a minor difference associated with smoking [35]. 

The dosing regimen for omadacycline varies depending on the prescribed indication and route of administration. Intravenous administration follows a uniform protocol across indications, with a loading dose of 200 mg once daily or 100 mg infusion administered twice, followed by a daily maintenance dose of 100 mg. In contrast, oral formulation regimens differ between CABP and ABSSSI. For CABP, patients receive an initial loading dose of 300 mg twice daily, followed by a once-daily maintenance dose of 300 mg. Conversely, in ABSSSI cases, the regimen involves a 2-day loading dose of 450 mg once daily, followed by a daily maintenance dose of 300 mg. The total duration of omadacycline administration typically spans 1 to 2 weeks [36]. 

## 6. Spectrum of Activity

### 6.1. Tigecycline 

Tigecycline exhibits broad-spectrum activity against various Gram-positive, Gram-negative, and anaerobic organisms, including MDR strains like methicillin-resistant *Staphylococcus aureus* (MRSA), methicillin-resistant *Staphylococcus epidermidis* (MRSE), penicillin-resistant *Streptococcus pneumoniae*, and vancomycin resistant *Enterococci* (VRE) species. In vitro studies show tigecycline’s improved efficacy compared to other tetracyclines against Gram-negative pathogens such as *Citrobacter freundii*, *Escherichia coli*, *Enterobacter cloacae*, *Klebsiella*, *Salmonella*, and Shigella species, and *Serratia marcescens* [37]. It also remains active against anaerobic bacteria like *Bacteroides fragilis*, *Bacteroides thetaiotaomicron*, *Bacteroides uniformis*, *Bacteroides vulgatus*, *Clostridioides difficile*, and *Clostridium perfringens*, with potential impact against *Mycobacterium abscessus* and *Mycobacterium fortuitum* [38,39,40,41].

However, like other tetracyclines, tigecycline lacks activity against certain pathogens like *Pseudomonas aeruginosa*, *Morganella morganii*, and *Providencia* spp. due to intrinsic resistance. It also does not cover certain *Proteus* strains, including *Proteus mirabilis* [15,36]. Compared to minocycline, tigecycline is less effective against methicillin-susceptible *Staphylococcus aureus* and *Staphylococcus epidermidis*, as well as against *Stenotrophomonas maltophilia* and *Burkholderia cepacia*. However, it remains more active against methicillin-resistant Staphylococcus strains [15]. Notably, both tigecycline and minocycline provide coverage against *Acinetobacter* species [37].

### 6.2. Eravacycline 

Eravacycline, in vitro, demonstrates broad-spectrum activity against both Gram-positive and Gram-negative aerobic and anaerobic pathogens, excluding *Pseudomonas aeruginosa*. Notable antimicrobial-resistant pathogens such as MRSA, VRE, ESBL- or carbapenemase-producing *Enterobacteriaceae*, and MDR *Acinetobacter baumannii* species are effectively targeted [42]. Eravacycline’s potency surpasses that of tigecycline against Gram-positive cocci by two- to fourfold and against Gram-negative bacilli by two- to eightfold [16]. Its robust activity and availability in both intravenous and oral forms position it as a viable alternative treatment for severe infections.

Studies highlight eravacycline’s efficacy against resistant Gram-positive pathogens, including its superiority to omadacycline against *S. aureus* [43]. In cases of infections caused by MDR pathogens, eravacycline shows promise, particularly against carbapenemase-producing Gram-negative bacilli strains and *Acinetobacter* species. Clinical data underscore eravacycline’s effectiveness against carbapenem-resistant Gram-negative bacteria, with superior bactericidal effects compared to tigecycline [31,44]. Real-world studies demonstrate its efficacy and safety in treating *Acinetobacter baumannii* infections, with low mortality rates and minimal drug-related adverse events [45,46]. Eravacycline also exhibits favorable outcomes against *Stenotrophomonas maltophilia* isolates, particularly resistant to levofloxacin and trimethoprim–sulfamethoxazole (TMP-SMZ) [47].

Recent global data from 2017 to 2020 reaffirm eravacycline’s advantages against both Gram-positive and Gram-negative bacteria. Notably, there are variations in susceptibility standards between the European Committee on Antimicrobial Susceptibility Testing (EUCAST) and the FDA, particularly for *Staphylococci* and vancomycin-resistant *Enterococcus faecalis* species. Nonetheless, eravacycline maintains comparable activity against MDR isolates of *Enterobacteriaceae*, *Acinetobacter baumannii*, and *Stenotrophomonas maltophilia* [48].

### 6.3. Omadacycline 

Omadacycline emerges as a noteworthy addition to the tetracycline class, showcasing broad-spectrum antimicrobial activity against various bacteria, including Gram-positive, Gram-negative, and atypical species. Its efficacy extends to challenging pathogens such as MRSA, penicillin- or macrolide-resistant *Streptococcus pneumoniae*, *β-hemolytic streptococci*, and VRE. While *Pseudomonas aeruginosa* may not be susceptible, omadacycline demonstrates in vitro activity against all ESKAPEE pathogens [49]. 

Data from the SENTRY Antimicrobial Surveillance Program underscore the beneficial effects of omadacycline in treating infections caused by Gram-positive pathogens. Omadacycline effectively inhibits a high percentage of *Staphylococcus aureus* isolates, including MRSA strains, as well as *Streptococcus pneumoniae*, viridans group streptococci, and beta-hemolytic streptococci. It also exhibits activity against *Enterobacterales*, with notable efficacy against *Escherichia coli*, *Klebsiella oxytoca*, and *Citrobacter* spp. However, its activity against *Proteus mirabilis* and indole-positive *Proteus* spp. is limited [50]. 

Regarding *Acinetobacter baumannii*, omadacycline’s effectiveness against carbapenem-non-susceptible (CNSAb) strains is promising, particularly when co-administered with sulbactam, achieving an 80% efficacy rate. However, its activity against extensively drug-resistant (XDR) *A. baumannii* strains may vary, with some strains showing intermediate or full resistance. Monotherapy with omadacycline in carbapenem-resistant *Acinetobacter baumannii* (CRAB) isolates demonstrates limited activity, with a synergistic effect observed in only a subset of isolates under high-dose intravenous daily exposures [51,52,53].

## 7. Resistance Mechanisms 

As regularly seen in practice, it is just a matter of time after the clinical application of newer drugs, such as the case of eravacycline and omadacycline in the tetracycline superfamily, for resistance to develop, but at lower percentages, by various mechanisms. Thus far, these known mechanisms regarding tetracyclines are the existence of efflux pumps, the inactivation of tetracyclines by several enzymes, the alterations in the target of tetracyclines, the decrease in outer membrane permeability, and the defective DNA repair mechanisms.

### 7.1. Efflux Pumps

Efflux pumps are specialized transport proteins that actively expel harmful substances, including antibiotics, from bacterial cells to the external environment, utilizing energy [54,55]. These pumps play a significant role in tetracycline resistance, with members of the major facilitator superfamily (MFS) being the most commonly encountered efflux pumps, although other non-MFS pumps have also been identified [54,55]. Among the extensively studied MFS members are Tet(A), Tet(B), and Tet(K) proteins [36,56]. It is worth noting that while these pumps were initially known for recognizing older tetracyclines, recent studies have shown that Tet(A), Tet(B), and Tet(K) also recognize newer agents like tigecycline, eravacycline, and omadacycline [14,36,57,58,59,60]. This delay in understanding their involvement in resistance mechanisms might be due to the introduction of eravacycline and omadacycline in clinical practice after tigecycline.

In addition to the major facilitator superfamily (MFS), various other efflux pumps have been identified, including ATP binding cassette (ABC) transporters, the multidrug and toxic compound multi-antimicrobial extrusion (MATE) family, the resistance nodulation cell division family (RND), and the small multidrug resistance family (SMR) [61,62,63]. ABC pumps utilize energy from ATP hydrolysis to expel tetracyclines from bacterial cells, while the MATE family relies on Na^+^ or H^+^ ions for its function [61,62]. RND pumps are prevalent among MDR *Acinetobacter baumannii* strains, with pumps like AdeABC, AdeFGH, and AdeIJK commonly implicated in tigecycline and eravacycline resistance [57,62,64]. Similarly, recent evidence has linked the drug efflux mechanism to tigecycline resistance in clinical isolates of *Klebsiella species*, including *K. pneumoniae*, *K. variicola*, and *K. michiganensis*, where RND-type efflux pumps like the TMexCD2-TOprJ2 clusters on IncHI1B plasmids are involved [57,65]. Furthermore, the small multidrug resistance (SMR) family has garnered attention at the molecular level due to its simple structure yet complex functions [57,63].

### 7.2. Enzymatic Inactivation

The main mechanism of tetracycline antibiotic inactivation involves the plasmid-mediated Tet(X) family, consisting of eight genes that encode enzymes conferring high-level resistance to tigecycline. The first described member, Tet(X), encodes a flavin-dependent monooxygenase that converts tigecycline into 11a-hydroxytigecycline, thereby weakening its binding to magnesium and ultimately to ribosomes [65,66,67]. Besides first- and second-generation tetracyclines and the glycylcycline tigecycline, resistance to eravacycline and recently omadacycline mediated by the Tet(X) family has also been documented [57,65,66,67]. The presence of the Tet(X) family on plasmids raises concerns due to its potential for widespread distribution, particularly among MDR strains of *A. baumannii* and *Enterobacterales* [68,69].

### 7.3. Modification in the Target of Action of Tetracyclines

As previously mentioned, tetracyclines exert their action by binding to the highly conserved 16S rRNA region of the 30S ribosomal subunit. Any modifications in this binding site can lead to reduced binding affinity and the development of resistance [57,70]. The *rpsJ* gene, encoding residues 53–60 in the S10 protein, is associated with the normal structure of the tigecycline binding site. Mutations in rpsJ have been linked to decreased binding affinity and resistance to tigecycline and eravacycline, particularly in *A. baumannii* [65]. Similarly, resistance to eravacycline has been observed in mutant *Enterococcus* species with mutations encoded by *rpsJ* gene [71]. In addition, the ribosome recycling factor (RRF) plays a crucial role in releasing polypeptides from the ribosome for initiation of a new translation cycle. Mutations in the *rrf* gene leading to decreased RRF production result in reduced translation cycling and ultimately tigecycline resistance, as evidenced in vitro in *A. baumannii* species [72]. Additionally, the *trm* gene encodes for S-adenosyl-L-methionine methyltransferase, an enzyme that methylates the target of tetracyclines, potentially reducing antibiotic binding to the ribosome [73]. Interestingly, omadacycline’s efficacy can be compromised by mutations in the ribosomal RNA of certain pathogens [74].

### 7.4. Decreased Outer Membrane Permeability

Mutations in genes responsible for the structure of porins have been linked to decreased outer membrane permeability in Gram-negative bacteria and mycobacteria. Porins are beta barrel proteins in the outer membrane that serve as channels for the passive diffusion of molecules [1,75,76]. Outer membrane proteins (OMPs), like OmpF and OmpC, have been implicated in tetracycline resistance [54]. Recently, tigecycline-resistant *Klebsiella pneumoniae* strains have been isolated, showing the differential expression of OmpK35 and OmpR [76]. Additionally, clinical isolates of carbapenem-resistant *Klebsiella pneumoniae* may rapidly develop resistance to eravacycline, partly due to the upregulation of porin proteins like OmpA and OmpU [76].

### 7.5. Defective DNA Repair Mechanisms

Ajiboye et al. have reported that defective DNA repair may be responsible for tigecycline resistance. In particular, they have described the involvement of RecA and RecBCD enzymes in gene repair mechanisms [77]. However, there is a lack of reports elaborating upon this specific issue.

**Figure 2:**
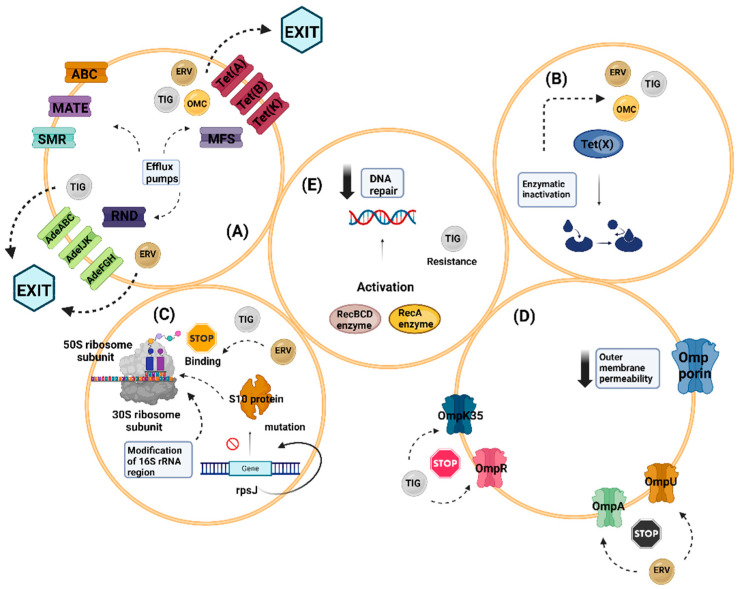
Main mechanisms of resistance to tetracyclines and third-generation analogs. (**A**) MFS and non-MFS (ABC, MATE, RND, and SMR) efflux pumps facilitate the removal of traditional and novel tetracyclines from bacterial cells to the external environment. (**B**) The plasmid-mediated Tet(X) family encodes enzymes that prevent third-generation tetracyclines from binding to ribosomes, resulting in resistance. (**C**) Mutations in the rpsJ gene, which encodes residues 53–60 in the S10 protein, result in the reduced binding affinity of third-generation analogs to their binding site on the 30S ribosome subunit. (**D**) Mutations in genes responsible for the structure of OMPs have been implicated in tetracycline resistance. (**E**) Activation of RecA and RecBCD results in an impaired DNA damage response in bacteria, particularly in *Acinetobacter baumannii* isolates [55,56,57,58,59,60,61,62,63,64,65,66,67,68,69,70,71,72,73,74,75,76,77]. Abbreviations: ABC, ATP binding cassette; ERV, eravacycline; MATE, multi antimicrobial extrusion; MFS, major facilitator superfamily; OMC, omadacycline; OMP, outer membrane proteins; RND, resistance nodulation cell division family; SMR, small multidrug resistance; TIG, tigecycline. Created with BioRender.com (accessed on 26 June 2024).

Overall, as more resistance mechanisms are yet to be discovered, the implication of efflux pumps together with enzymatic inactivation of the tetracycline superfamily and modifications in the tetracyclines’ target still remain the major resistance mechanisms of these molecules. Nevertheless, we should not overlook the fact that different resistance mechanisms may be involved in different species but may also co-exist within the same strain. In this context, the constant improvement in molecular diagnostics is anticipated to shed light upon novel resistance mechanisms. Figure 2 summarizes the main mechanisms of resistance to tetracyclines and their third-generation analogs. 

## 8. Established Indications of Prescription 

### 8.1. Tigecycline 

Tigecycline was initially granted approval by the FDA in 2005 and later by the European Medicines Agency (EMA) in 2006. Its approval was primarily for the treatment of cIAIs and cSSTIs. Notably, tigecycline’s effectiveness and safety in treating cIAIs were underscored by favorable outcomes when compared to the use of imipenem–cilastatin [78]. Similarly, in the context of cSSTIs, tigecycline demonstrated non-inferiority to the clinical responses observed with the vancomycin–aztreonam combination, with comparable adverse events between the two antibiotic regimens [78]. In 2009, the FDA expanded tigecycline’s approved indications to include CABP. This decision followed compelling evidence from studies suggesting that tigecycline achieved similar cure rates to levofloxacin, while maintaining a favorable safety profile [79].

### 8.2. Eravacycline 

Eravacycline received FDA approval in 2018 for treating adults with complicated intra-abdominal infections (cIAIs), based on results from two Phase III multicenter randomized controlled trials (RCTs), IGNITE 1 and IGNITE 4. These double-blind trials showed that eravacycline’s efficacy was comparable to ertapenem and meropenem in cIAI patients [80,81]. Additionally, a recent Bayesian network meta-analysis compared eravacycline’s efficacy and safety with commonly used antibiotics in adults with cIAIs. Eravacycline showed comparable clinical response rates to other therapies and superiority to tigecycline in microbiological response rates. Safety outcomes, including serious adverse events, the discontinuation rate, and all-cause mortality, did not differ significantly from other therapies [82]. The effectiveness and safety of eravacycline were also assessed in IGNITE2 and IGNITE3 trials for treating cUTIs compared to levofloxacin [82,83,84]. While eravacycline did not achieve statistical non-inferiority compared to levofloxacin at the post-therapy visit in the micro-ITT population, it demonstrated higher responder rates at the end of therapy. Interestingly, eravacycline showed higher response rates compared to levofloxacin in patients with quinolone-resistant pathogens and in those who received at least 7 days of eravacycline treatment [82,83,84].

### 8.3. Omadacycline 

In 2018, the FDA approved the use of omadacycline in adults with CABP and ABSSSI caused by susceptible pathogens. In a post-hoc analysis of the phase 3 OPTIC trial, omadacycline demonstrated comparable efficacy to moxifloxacin in treating CABP among subjects classified as Pneumonia Severity Index (PSI) risk class II/III with comorbidities [85]. Interestingly, omadacycline emerges as a viable treatment option for individuals with complicated skin and soft tissue infections. A recent meta-analysis, incorporating findings from four randomized controlled trials involving 1757 subjects, revealed that omadacycline demonstrated comparable safety, efficacy, and microbiological response to linezolid in this context. Importantly, both treatment groups exhibited similar rates regarding side effects and mortality [86]. 

## 9. Potential Indications of Administration 

### 9.1. Mycobacterial Infections 

Accumulating evidence demonstrates that third-generation tetracyclines are effective against infections caused by the *Mycobacterium abscessus* complex (MABC). The MABC comprises rapidly growing, non-tuberculous, multidrug-resistant mycobacteria associated with severe pulmonary, skin, and skin structure infections, posing a high mortality risk for immunocompromised patients. Notably, *Mycobacterium abscessus* lung disease presents significant challenges due to its high resistance to conventional antibiotics. Tigecycline is effective against rapidly growing mycobacteria like *M. fortuitum*, *M. chelonae*, and *M. abscessus* but lacks activity against slower-growing mycobacteria such as *M. tuberculosis* [87,88]. Tigecycline-containing regimens have shown high rates of symptomatic and radiological improvement in *M. abscessus* disease [89]. 

Eravacycline has also shown promise as a treatment for non-tuberculous mycobacteria (NTM) infections, particularly against *M. abscessus*. Studies reveal its efficacy against various non-*M. abscessus* NTM species, including *M. chelonae* and *M. immunogenum*. Experimental studies with clinical NTM isolates indicate that eravacycline is effective against all rapidly growing mycobacteria (RGM) species, with MIC50 ranges supporting significant inhibition. These findings suggest that eravacycline could be a valuable addition to NTM treatment options, especially for these species [90,91]. Similar observations have been reported for omadacycline, whose activity against various *M. abscessus* strains appears to be comparable to or even superior to tigecycline. After a seven-day in vitro exposure, omadacycline demonstrated concentration-related anti-*M. abscessus* activity, with bacteriostatic effects at 4 μg/mL and bactericidal effects at concentrations exceeding 16 μg/mL. Importantly, no evidence of inducible resistance was detected [92]. 

In a multicenter retrospective study involving 75 patients from 16 medical institutions in the United States, the long-term effects of omadacycline on NTM infections were examined, focusing on efficacy, safety, and tolerability. The majority of patients (44.0%) had NTM lung disease, with *Mycobacterium abscessus* being the most frequently isolated NTM pathogen (80%). The study reported a 3-month clinical success rate of 80.0% among patients treated with omadacycline. Adverse events related to omadacycline occurred in 32.0% of patients, leading to drug discontinuation in 9.3% of cases [93]. 

Moreover, some studies have focused on omadacycline’s potential as a therapeutic option for tuberculosis, particularly in cases of MDR isolates. In a study involving *Mycobacterium tuberculosis* (Mtb) H37Rv and clinical strains of MDR-TB, omadacycline exhibited an MIC of 16 mg/L. However, this MIC decreased to 4 mg/L when considering omadacycline degradation with daily drug supplementation [94].

### 9.2. Clostridioides Difficile Infection 

Tigecycline has shown significant in vitro activity against *C. difficile* [95]. The European guidelines published in 2014 advocated the use of the drug for severe and/or severely complicated or refractory *C. difficile* infection (CDI) as a salvage therapy when the administration of oral therapy is not feasible [39]. A meta-analysis in 2020 provided further supportive evidence for the potential role of tigecycline in the treatment of patients with CDI [39]. On the contrary, a 2022 case series and propensity-matched cohort study showed that tigecycline did not significantly improve 30-day mortality [96]. 

Preliminary studies show promise for eravacycline in treating CDIs. In an in vitro human gut model, eravacycline did not induce CDI, despite initially affecting the intestinal microbiota [97]. On the contrary, the drug has been shown to exhibit bactericidal activity against various *C. difficile* strains, including epidemic RT027, irrespective of vancomycin susceptibility or resistance gene presence [98].

Similarly to the other third-generation tetracyclines, omadacycline has been reported to demonstrate in vitro efficacy against *C. difficile*, with clinical trials reporting low CDI rates compared to other antimicrobials for CABP and ABSSSI. The drug seems to maintain a high activity level against *C. difficile* strains, supported by stable minimum inhibitory concentrations among contemporary isolates [99]. Additionally, omadacycline shows promise in preventing CDI relapse, outperforming vancomycin in murine models [99]. A phase I trial in healthy participants found omadacycline to be safe, effective, and well tolerated, with rapid fecal concentration increase compared to vancomycin (VAN) [100]. 

### 9.3. Infection from Helicobacter pylori 

Existing evidence suggests that tigecycline exhibits robust in vitro activity against *Helicobacter pylori* isolates, with an MIC90 of 0.06 μg/mL [101]. In a human study involving 111 participants from whom 91 *H. pylori* strains were isolated, tigecycline demonstrated the lowest resistance rates (up to 5%) compared to commonly prescribed antibiotics for *H. pylori* infection treatment, including amoxicillin, clarithromycin, levofloxacin, and metronidazole [102]. Additionally, eravacycline along with omadacycline may present promising treatment options for tetracycline-resistant *H. pylori* strains. Among 201 clinical isolates of *H. pylori*, both omadacycline and eravacycline exhibited superior in vitro efficacy compared to tetracycline [103,104].

### 9.4. Urinary Tract Infections 

While some case reports have suggested benefits in treating urinary tract infections (UTIs) with tigecycline, its use remains controversial due to the lack of evidence regarding its efficacy from randomized control trials [105]. However, a recent systematic review revealed favorable clinical (77.4%) and microbiological (65.2%) outcomes following tigecycline administration in individuals with complicated urinary tract infections (cUTIs), except in cases where the underlying pathogen was *K. pneumoniae*. The high success rates may be attributed to the broad spectrum of activity of tigecycline, along with its ability to render highly resistant Gram-negative bacteria, including extended spectrum beta-lactamase (ESBL) or carbapenem-resistant Enterobacterales (CRE), sensitive even at low concentrations [106]. Nevertheless, tigecycline cannot be considered the primary therapeutic approach in such cases, wherein well-established treatment options like β-lactams and aminoglycosides should be preferred. Instead, tigecycline may be used alternatively in cases where it is the only susceptible drug [107].

On the other hand, omadacycline with an MIC of ≤4 µg/mL may offer a promising oral treatment option for UTIs caused by ESBL-producing *Enterobacterales*, especially for *E. coli* isolates. It has been observed to be effective in 74.5% of cases compared to 54.9% for *K. pneumoniae*, respectively [108]. Table 2 presents recent studies that elucidated the potential impact of third-generation analogs on CDI, MABS, and *H. pylori* infection. 

## 10. Synergistic Benefits in Combination Therapy

Third-generation tetracyclines have undergone extensive testing across a wide spectrum of studies exploring their potential synergistic role in treating severe infections, particularly those caused by drug-resistant Gram-negative bacteria. However, recent research has shed light on the potential efficacy of third-generation tetracyclines, particularly omadacycline, as an additional regimen for severe infections caused by Gram-positive microorganisms. Omadacycline, when combined with rifampicin, has emerged as a promising alternative treatment for cases of MRSA osteomyelitis [110]. Preliminary data indicate that the co-administration of omadacycline with rifampicin may be an effective approach for biofilm-associated *Staphylococcus aureus* and *Staphylococcus epidermidis* strains, resulting in prompt and sustained bactericidal activity across almost all strains. Furthermore, the addition of omadacycline has shown benefits in preventing rifampicin resistance [111].

Third-generation tetracyclines could emerge as potent treatment options for combating organisms harboring ESBL and CRE. In vivo and in vitro data suggest that tigecycline, when combined with aminoglycosides, may exhibit synergistic properties against carbapenem-resistant *Klebsiella pneumoniae (CRKP)* species, thereby reducing the emergence of tigecycline-resistant mutants [112]. Among 49 non-duplicate CRKP strains, additional activity was observed in 75.5% of isolates for tigecycline–amikacin and 69.4% for tigecycline–gentamicin [113]. The proposed hypothesis suggests that aminoglycosides induce mistranslation of amino acids, leading to adverse impacts on the bacterial cytoplasmic membrane and disrupting the protein synthesis process. These translation errors not only compromise protein synthesis but also reduce β-lactamase expression, directly impacting aminoglycoside activity. Consequently, the synergistic effects of combined antibiotics are expected to be potentiated by aminoglycosides at target sites [114]. Additionally, the combination of antibiotics could potentially reduce mutational frequencies, thereby mitigating the development of resistant genes [115]. The recommended combination dose regimen includes the initial use of 200 mg tigecycline and 25 mg/kg amikacin or 7 mg/kg gentamicin, followed by 100 mg tigecycline every 12 h and 15 mg/kg amikacin or 5 mg/kg gentamicin every 24 h [116]. Emerging evidence highlights the role of eravacycline, as this drug demonstrates synergistic advantages when paired with cephalosporins or polymyxin B. Notably, eravacycline combined with polymyxin B exhibits remarkable efficacy against *Escherichia coli* isolates, achieving a synergism rate of 60%. Conversely, the combination of eravacycline with ceftazidime proves highly effective against *Acinetobacter baumannii*, exhibiting an impressive 80% synergism rate [117]. 

Tigecycline-based combinations are gaining traction in the treatment of serious CNSAb infections due to their robust in vitro activity, synergistic potential when combined with other agents, and favorable toxicity profile. The impact of tigecycline-based combinations has been demonstrated in various studies, showing that tigecycline combined with β-lactams, carbapenems, or polymyxin B can lead to high synergistic activity against carbapenem-resistant or multidrug-resistant *A. baumannii* isolates [118]. On the other hand, evidence concerning eravacycline is limited, with data showing that when eravacycline is combined with colistin against ten carbapenem-resistant *A. baumannii* isolates, it exhibits 10% synergy and no antagonism [119]. Recent data suggest more favorable outcomes, as eravacycline combined with ceftazidime or polymyxin B can lead to a synergistic effect against more than 50% of carbapenem-resistant *Acinetobacter baumannii* [120]. Furthermore, evidence suggests that omadacycline may have in vitro advantages over existing tetracycline derivatives. Certain antibiotic combinations, mainly omadacycline with sulbactam, may provide significant results regarding effectiveness against CNSAb infections. Omadacycline monotherapy may be ineffective, whereas sulbactam has been shown to be effective against only 10% of isolates. However, the landscape is reversed in cases of co-administration of omadacycline with sulbactam, as this dual-drug combination exhibits synergy, showing effectiveness against 80% of isolates. This combination regimen seems superior compared to amikacin and polymyxin B, which exert synergy against 30% of isolates [120].

Emerging evidence suggests that omadacycline may enhance the activity of clarithromycin against *Mycobacterium abscessus* [120]. Moreover, omadacycline may promote in vitro synergistic properties with other antibiotics against MAB, with a tendency to be more beneficial against rough-morphotype strains. Among different antimicrobial agents, the co-administration of omadacycline with rifampicin was identified as the most effective combination regimen, resulting in an efficacy rate of 76.9% [121]. In addition, omadacycline prescription has been shown to be efficient against strains with a high level of resistance to various antibacterial agents, with the potential to boost the effects of macrolides and linezolid against several isolates. In a mouse model of *Mycobacterium abscessus* lung disease, combination therapy with omadacycline and cefoxitin, linezolid, carbapenem, or rifabutin demonstrated early bactericidal activity during the initial phase of treatment [122]. Regarding tigecycline, the co-administration with teicoplanin has been reported to lead to synergy against 70.4% of the *M. abscessus* isolates, representing the three subspecies of *M. abscessus*, with growth inhibitory combination concentrations of 2–3 μM teicoplanin +1–2 μM tigecycline [123]. On the contrary, eravacycline has not shown either synergistic or antagonistic effects when combined with rifabutin and clarithromycin [124].

Interestingly, the combination of third-generation tetracyclines with fluconazole holds promise for combating *Candida species* infections, although the precise mechanisms are yet to be fully understood. Studies have demonstrated in vitro synergy with *C. albicans* biofilms when using tigecycline in combination with fluconazole [125]. Notably, this combination exhibits a remarkable 94% synergy rate, significantly outperforming the synergy observed with doxycycline plus fluconazole, where only 28% of isolates exhibited synergy [126]. Additionally, eravacycline has shown potential synergistic effects when combined with fluconazole in treating resistant *Candida albicans* species, both in vitro and in vivo. This synergy is believed to arise from the inhibition of DNA replication and cell meiosis [127].

## 11. Non-Antibiotic Properties: Focusing on Immunomodulation and Malignancy

While traditionally utilized for their broad-spectrum antibiotic properties, tetracyclines have garnered attention for their non-infectious features. While minocycline has been extensively studied in this regard, third-generation tetracyclines have demonstrated intriguing immunomodulatory capabilities, influencing various aspects of the immune response beyond their antibiotic activity. Additionally, mounting evidence suggests their potential utility in the management of certain malignancies, offering novel therapeutic perspectives beyond their antimicrobial role. The bulk of research on the non-infectious effects of third-generation tetracyclines has centered on tigecycline. Limited data exist regarding the immunomodulatory effects of eravacycline and the potential role of omadacycline in malignancy, respectively. 

### 11.1. Immunomodulation 

**Figure 3:**
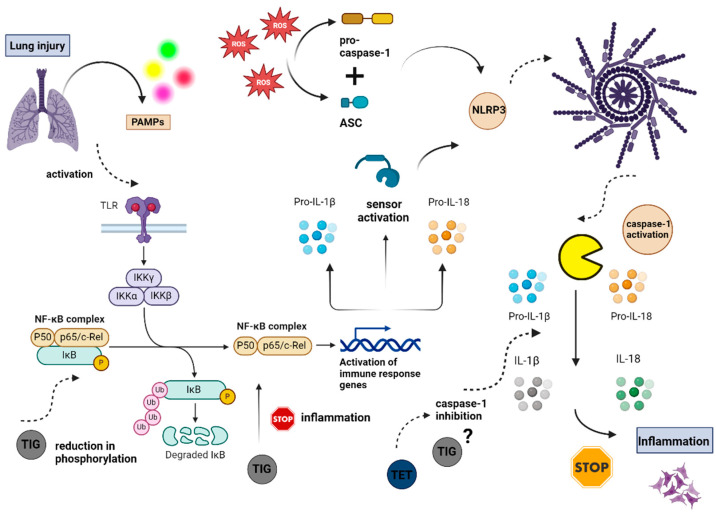
Schematic illustration of the potential mechanisms by which tetracyclines and tigecycline may interfere with the inflammatory response via NLRP3 inflammasome. Acute pulmonary infection triggers the release of PAMPs, which stimulate TLRs and subsequently activate NF-κB. The phosphorylation of NF-κB induces the transcription of genes encoding pro-IL-18 and pro-IL-1β cytokines, leading to the polymerization and activation of the NLRP3 receptor. Tigecycline reduces NF-κB phosphorylation, thereby mitigating the inflammatory response. Hence, tigecycline may attenuate the inflammatory process by intervening at an earlier stage before the formation of NLRP3. In contrast, tetracyclines act at a different point in this pathway by inhibiting caspase-1 to alleviate inflammation. Despite these differences, both tetracyclines and tigecycline weaken the inflammatory response and are considered anti-inflammatory agents [129,130,131]. Abbreviations: ASC, apoptosis-associated speck-like protein containing a CARD domain; ΙκΒ, IκB kinase; IL-1β, interleukin-1β; IL-18, interleukin-18; NF-κΒ, nuclear factor-kappa-light-chain-enhancer of activated B cells; NLRP3, nucleotide-binding domain, leucine-rich-containing family, pyrin domain-containing-3 inflammasome; PAMPs, pathogen-associated molecular patterns; Pro-IL-1β, pro-interleukin-1β; Pro-IL-18, pro-interleukin-18; TET, tetracyclines; TIG, tigecycline; TLR, toll-like receptor. Created with BioRender.com (accessed on 26 June 2024).

Preclinical data from animal models of bacterial infection have shown that tigecycline may downregulate the expression of LPS-induced inflammatory molecules by diminishing nuclear factor-kappa beta (NF-κB) phosphorylation and by mitigating phosphorylation of p38 and activation of the ERK1/2 pathway. This leads to a significant reduction in the expression of pro-inflammatory molecules such as tumor necrosis factor-alpha (TNF-α), IL-8, macrophage inflammatory protein 1α (MIP-1α), and MIP-1β by LPS-stimulated THP-1 cells [128]. Similarly, recent research has recognized tetracyclines for their immunomodulatory properties, particularly their ability to directly inhibit the secretion of pro-inflammatory cytokines IL-1β and IL-18 via the NLRP3 inflammasome–caspase-1 pathway [129]. Given that the phosphorylation of NF-κB is pivotal in the NLRP3 inflammasome formation, tigecycline, or even novel third-generation analogs, may provide significant anti-inflammatory effects through this pathway. This is of particular importance, as this pathway is highly involved in the host immunity against clinically relevant *A. baumannii* lung infection [130]. The beneficial role of tigecycline in severe pulmonary infection and its potential anti-inflammatory effects via the NLRP3 inflammasome–caspase-1 pathway are illustrated in Figure 3. However, further research is needed to elaborate on this issue.

Furthermore, the ability of tigecycline to intervene in the inflammatory process may explain its potential neuroprotective role. Yagnik et al. demonstrated that tigecycline may impede the LPS-induced release of pro-inflammatory and apoptotic mediators in neuronal cells. Their study results showed that in LPS-induced PC12 cells, tigecycline significantly reduced both the release and the expression of NF-κB, TNF-α, and IL-1β, with beneficial effects on nitric oxide (NO) levels as well. Moreover, in a dose-dependent manner, tigecycline reduced caspase-3 activity, confirming the results of reduced pro-apoptotic Bad, and enhanced anti-apoptotic Bcl-2 protein expression [131]. 

Interestingly, tigecycline seems to enhance the pro-inflammatory functions of human neutrophils in vitro. Preliminary experimental data suggest that tigecycline infusion mitigated oxidative stress, with the fundamental involvement of the increment of cytosolic Ca^2+^, due to its ability to act as a Ca^2+^ ionophore [132]. However, while safe at therapeutic levels, higher doses may negatively impact the immune system, altering non-specific immune response and T cell function. Specifically, elevated doses can enhance IL-2 production while reducing IL-17 secretion. IL-2, produced by Th-1 cells, stimulates macrophages against intracellular antigens and inhibits IL-4/Th-2, promoting Th-1 and Treg development. This elevation may suppress IL-17, crucial for Th-17 development. These findings may explain the FDA’s warning regarding increased mortality rates post-tigecycline administration [133]. 

On the other hand, data on the immunomodulatory effects of eravacycline are lacking, while information on omadacycline is limited but promising. Omadacycline has shown potential in mitigating cytokine-mediated tissue injury by dampening hyperactive immune responses, which could improve overall clinical outcomes. This effect is particularly notable when pro-inflammatory M-type 1 macrophages dominate the immune response. A recent human study has highlighted omadacycline’s ability to reduce the production of pro-inflammatory molecules induced by *Escherichia coli* LPS. These include TNF-α and IL-1β, acute-phase reactants like IL-6, and anti-inflammatory cytokines (IL-4, IL-10), as demonstrated in vitro using primary human monocytes. Omadacycline effectively decreased LPS-induced cytokine production in a dose-dependent manner, especially at concentrations exceeding 32 μg/mL. Importantly, this effect was not due to drug cytotoxicity, as omadacycline both alone or in combination with LPS did not result in significant cell loss or apparent cytopathic changes [134]. 

### 11.2. Malignancy 

Emerging evidence across various hematologic malignancies and solid tumors highlights the diverse positive effects of tigecycline treatment. Tigecycline has been identified for its anti-cancer effects in subjects with acute myeloid leukemia (AML) due to its ability to inhibit mitochondrial translation [135]. Combining c-Abl-specific tyrosine kinase inhibitors (TKIs) with tigecycline emerges as a promising strategy for chronic myeloid leukemia (CML) treatment. Furthermore, tigecycline use holds promise for hematological malignancies such as acute lymphoblastic leukemia and diffuse large B-cell lymphomas [136,137]. Tigecycline’s efficacy extends to various solid tumors including gastric cancer, oral squamous cell carcinoma, melanoma, neuroblastoma, and glioma. Additionally, the drug may offer benefits in cases of triple-negative breast cancer, lung cancer, prostate cancer, pancreatic cancer, cervical squamous cell carcinoma, ovarian cancer, and hepatocellular carcinoma [136,137,138,139,140]. 

**Figure 4:**
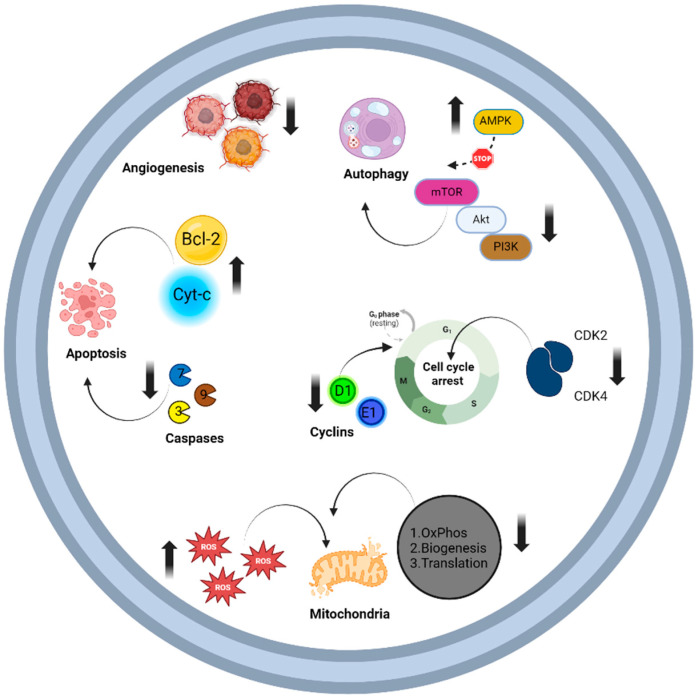
Schematic presentation of the effects of third-generation tetracyclines, particularly tigecycline in tumor cells. Tigecycline affects programmed cell death pathways, including autophagy and apoptosis. It promotes autophagy by activating the AMPK pathway, which inactivates mTOR, and by downregulating the PI3K-AKT-mTOR pathway. Tigecycline facilitates apoptosis through the activation of BCL-2 and the release of cytochrome c, and by enhancing the cleavage of caspase-3, caspase-7, and caspase-9. In mitochondria, tigecycline induces oxidative injury, suppresses oxidative phosphorylation, and inhibits mitochondrial biogenesis. Additionally, it promotes cell cycle arrest, affecting the proliferation of cancer cells through alterations in cyclins and cyclin-dependent kinase levels. Lastly, tigecycline exhibits anti-angiogenic properties [135,136,137,138,139,140,141]. Abbreviations: Akt, serine/threonine kinase 1; AMPK, adenosine monophosphate-activated protein kinase; BCL-2, B-cell lymphoma 2; CDK, cyclin-dependent kinase; mTOR, mammalian target of rapamycin; OxPhos, oxidative phosphorylation; PI3K, phosphatidylinositol-3 kinase; ROS, reactive oxygen species. Created with Biorender.com (accessed on 26 June 2024).

Tigecycline primarily influences malignancy by reducing cell proliferation and aerobic metabolism while promoting cellular apoptosis, oxidative stress, angiogenesis, autophagy, and mitochondrial dysfunction in tumor cells. Notably, combining tigecycline with chemotherapeutic or targeted agents such as venetoclax, doxorubicin, vincristine, paclitaxel, cisplatin, and imatinib has demonstrated encouraging synergistic effects in cancer treatment [136,137,138,139,140,141]. In a murine experimental model simulating colitis-associated colorectal cancer (CAC), tigecycline demonstrated significant antiproliferative effects by targeting the Wnt/β-catenin pathway and inhibiting STAT3 activity. This is particularly relevant considering that around 80% of colorectal malignancies exhibit APC gene mutations, leading to aberrant β-catenin accumulation and uncontrolled cellular proliferation. Tigecycline’s ability to trigger apoptosis through various pathways, resulting in elevated CASP7 levels, has shown promise in reducing inflammation primarily by decreasing cytokine expression [142,143,144]. Figure 4 presents a schematic overview of the primary mechanisms underlying the anti-cancer properties of tigecycline.

Recent research unveils eravacycline’s potential in treating pancreatic ductal adenocarcinoma (PDAC), indicating the scope for repurposing drugs in cancer therapy through machine learning techniques. Eravacycline inhibits tumor cell proliferation and migration while promoting cancer cell apoptosis, exhibiting significant dose-dependent reduction in BxPC-3 cell proliferation compared to tigecycline or omadacycline. However, its efficacy appears less pronounced in other cancer cell lines such as breast (MCF-7), lung (A549), and colon (HT-29) cancers [145]. Notably, eravacycline could serve as a therapeutic option for bacterial infections in cancer patients, as it shows effectiveness against various clinically significant bacteria, including MRSA, carbapenem-resistant *Enterobacterales*, and non-fermenting Gram-negative bacilli [146]. The remarkable effects of tigecycline, along with the promising results of eravacycline on malignancy, could guide the investigation of similar potential advantages regarding omadacycline as well.

## 12. Highlighting the Future Perspectives of Third-Generation Tetracyclines

Third-generation tetracyclines have emerged as crucial tools in combating MDR bacteria. In the face of increasing challenges posed by MDR *Acinetobacter baumannii* infections, these compounds show considerable promise [37,45,46,52,53]. They also offer significant utility in treating MDR non-tuberculous mycobacterial infections, particularly threatening to immunocompromised patients [88,89,90,91,92,93]. Additionally, there is growing optimism regarding their effectiveness against infections caused by *C. difficile* and *H. pylori* [96,99,102,103,104]. Omadacycline, developed for oral administration, stands out for its potential efficacy against CABP and ABSSSI [85,86]. Moreover, when combined with other antimicrobial classes such as β-lactams or aminoglycosides, third-generation tetracyclines demonstrate synergistic effects [113,114,117]. This synergism is particularly valuable in addressing MDR infections, where multiple mechanisms of resistance are encountered. Incorporating third-generation tetracyclines into our arsenal against MDR bacteria addresses critical unmet needs in infectious disease management. 

Furthermore, experimental studies underscore not only their role as antibiotics but also their potential as modulators of the immune response [129,130,131,132,133,134]. This dual action holds significant implications for treating both inflammatory and infectious diseases. In addition, tetracyclines, especially tigecycline among its third-generation analogs, exhibit promising anti-cancer properties [135,136,137]. The emerging potential of eravacycline further advocates for the continued exploration and optimization of tetracycline antibiotics in cancer treatment regimens [145,146]. Future research should focus on fully elucidating the immunomodulatory and anti-cancer effects of third-generation analogs and optimizing their therapeutic application.

## 13. Side Effects of Third-Generation Tetracyclines 

Over the years, a plethora of side effects stemming from both clinical trials and real-world usage of tetracyclines have been extensively documented. These adverse effects, characteristic of the tetracycline class, are commonly observed in most cases of third-generation tetracycline administration. Among them, gastrointestinal adverse events such as nausea, vomiting, diarrhea, and gastrointestinal discomfort prevail, typically following a dose-dependent pattern [147,148,149]. In some instances, additional symptoms like anorexia, constipation, and acute pancreatitis may manifest [150]. Third-generation tetracyclines have higher rates of acute pancreatitis and gastrointestinal side effects, such as diarrhea, vomiting and abdominal distention, compared to second-generation tetracyclines [17,149,150]. However, second-generation tetracyclines, like doxycycline, are more commonly linked to esophagitis [151]. 

Although comparative data are limited, it appears that eravacycline exhibits a better tolerability profile than tigecycline, with tetracycline class effects being common to both agents [150]. Moreover, third-generation tetracyclines are contraindicated in early childhood and during pregnancy due to their potential to induce tooth discoloration, enamel hypoplasia, and hindered bone growth. These effects are attributed to the formation of stable complexes between these tetracyclines and calcium ions, leading to their accumulation in deposits at these sites, resulting in the potentially permanent yellow to brown discoloration of teeth [151].

Individuals undergoing tetracycline treatment may experience infusion site reactions and allergic-type reactions such as pruritus, transient rash, or itching. Additionally, hyperhidrosis may occur, and in rare cases, hypersensitivity reactions such as Stevens–Johnson syndrome may be observed [152]. Third-generation tetracyclines have also been associated with central nervous system symptoms, including headache, insomnia, and dizziness, particularly following omadacycline administration and less commonly after eravacycline use [153]. Regarding cardiovascular side effects, omadacycline may promote arterial hypertension and increased heart rate due to the inhibition of carbamylcholine binding to the M2 subtype of the muscarinic acetylcholine receptor. However, omadacycline has a low potential for triggering cardiac arrhythmias or clinically significant cardiovascular toxicity, with observed increases in heart rate tending to decline over time and not reaching clinical significance. Among the tetracyclines, omadacycline uniquely interacts with the M2 receptor. Consequently, second-generation tetracyclines, as well as tigecycline and eravacycline, do not exhibit cardiotoxicity [154].

Following the prescription of third-generation tetracyclines, a diverse array of abnormalities may be observed on laboratory evaluation. Tetracyclines have been associated with elevations in serum creatinine and urea nitrogen levels, as well as abnormalities in liver enzymes, including elevations in serum aminotransferases, γ-glutamyl transferase, alkaline phosphatase, and bilirubin. Additionally, they may result in elevations in creatine phosphokinase levels [155]. Particularly, tigecycline and omadacycline may induce anemia. Tigecycline has been associated with thrombocytopenia, while omadacycline may lead to thrombocytosis [156,157]. Furthermore, tigecycline administration may prolong partially activated thromboplastin time (aPTT) and prothrombin time, with minimal impact on the international normalized ratio (INR) [158]. While rare, tigecycline administration may lead to hypoglycemia, irrespective of the presence of type 2 diabetes mellitus, which can occur at any time during tigecycline administration and may persist for days after discontinuation of the medication. Among predisposing factors, renal impairment and kidney replacement therapy are significant contributors [159,160,161].

Based on evidence from phase 3 and 4 trials, tigecycline carries a warning of the risk of an increase in all-cause mortality relative to comparators; however, the cause of this increase has not been established. Therefore, when selecting among treatment options, this potential increase in all-cause mortality seen with tigecycline therapy should be carefully considered [160,161]. Similarly, omadacycline administration has been correlated with increased mortality rates. Clinical trials evaluating its use in CABP have shown an imbalance in mortality between the two treatment groups, with death occurring in 2% of the omadacycline group compared to 1% of the moxifloxacin group. However, the reason for this mortality imbalance remains unknown [151]. Nevertheless, we should bear in mind that third-generation tetracyclines, such as tigecycline, are mainly used for severe infections due to MDR bacteria. Moreover, third-generation tetracyclines, such as omadacycline and eravacycline, have gained FDA approval in 2018, i.e., relatively recently and adverse effects have not yet been fully described. Overall, apart from this increased all-cause mortality, particularly with tigecycline, third-generation tetracyclines are well tolerated with mild and tolerable adverse effects in the clinical setting [162]. 

## 14. Conclusions

Despite the introduction of numerous novel antibiotic agents in recent years, tetracyclines remain a popular treatment choice due to their broad spectrum of activity. However, the widespread use of tetracyclines has led to the emergence of resistant strains, particularly in hospital settings, prompting the development of third-generation tetracyclines. Notably, unlike traditional tetracyclines, which are typically bacteriostatic, in vitro evidence suggests that these novel tetracyclines may exhibit bactericidal activity. About fifteen years ago, tigecycline received its first therapeutic indication, followed more recently by eravacycline and omadacycline. Third-generation tetracyclines have demonstrated significant therapeutic efficacy and are now pivotal in managing MDR hospital infections, often as part of combination therapies with other antibiotics. Additionally, they have shown promising results in treating other serious hospital-related infections, such as CDI and infections caused by MABS, although they are not yet considered established treatment options for these conditions. Moreover, experimental data, primarily concerning tigecycline, indicate that these drugs may positively impact inflammation and malignancy. This is clinically significant, as cancer patients often face life-threatening infections. It would be interesting to see future studies extend the investigation of tetracyclines’ effects on inflammation and malignancy to novel analogs such as eravacycline and omadacycline.

## Figures and Tables

**Table 1:** Classification of Tetracyclines [11].

First Generation	Second Generation	Third Generation
Naturally synthetized	Semi-chemical derivatives	Fully synthesized analogs
1. Tetracycline 2. Chlortetecycline 3. Oxytetracycline 4. Demeclocycline	1. Doxycycline 2. Lymecycline 3. Meclocycline 4. Methacycline 5. Minocycline 6. Rolitetracycline	1. Tigecycline 2. Eravacycline 3. Omadacycline 4. Sarecycline

**Table 2:** Evidence and concerns regarding potential future indications for third-generation tetracyclines in clinical practice.

Author, Year	Agent	Purpose/Characteristics	Main Findings/Remarks
Bassères, 2020 [98]	ERV	1. Evaluation of in vitro activity of ERV and FDX, VAN, MTZ against 6 common *C. difficile* ribotypes (234 strains), including isolates with ↓ VAN/MTZ susceptibility 2. Additionally tested: - MBCs - Time-kill kinetics - WGSs	1. Robust in vitro activity of ERV against *C. difficile* isolates 2. ERV’s efficacy was not affected by: - Ribotype - Susceptibility to VAN - ERV’s MIC was not influenced by the presence of tetM or tetW resistance genes 3. ↓ MIC50/90 values for ERV: - ERV: ≤0.0078/0.016 mg/L - FDX: 0.016/0.063 mg/L - MTZ: 0.25/1.0 mg/L - VAN: 2.0/4.0 mg/L 4. MBCs were ↓ for ERV vs. VAN for all ribotypes tested 5. Both ERV and VAN exhibited bactericidal killing at 8×, 16× and 32× the MIC, including epidemic RT027
Yang, 2020 [104]	ERV, OMC	Comparison of in vitro activity of ERV and OMC vs. TET against 201 isolates of *H. pylori* retrieved from biopsy samples from subjects with gastritis or gastric cancer	1. ERV and OMC are potent in vitro against *H. pylori* strains: - ERV vs. TET: ↑ eightfold potency - OMC vs. TET: ↑ fourfold potency 2. ERV’s and OMC’s potency are unaffected by the TET resistance: - 6 out of 201 isolates were TET-resistant with MICs of ≥2 μg/mL - All 201 isolates had ERV and OMC MICs of ≤1 μg/mL - TET-resistant strains showed ↓↓ ERV MICs (0.063 to 0.25 μg/mL) and OMC MIC (0.125 to 1 μg/mL) values
Phillips, 2021 [96]	TIG	1. Retrospective cohort of 28 CDI cases treated with TIG 2. Evaluation of the effect of TIG use on 90-day mortality and recurrency 3. In all cases, TIG was injected in combination with oral VAN +/− MTZ with a mean duration of treatment at 7.6 days	1. Patients treated with TIG showed ↑ in-hospital mortality, particularly when suffering from fulminant disease - 90-day mortality in 35.7% of the subjects - 50% mortality rates in fulminant infection 2. ↑ rate of CDI recurrency: 43.8% of surviving patients that reached 90-day follow-up had recurrent *C. difficile* infection
Kim, 2022 [109]	TIG	1. Evaluation of subjects with *M. abscessus* PD treated with multidrug regimens 2. Comparison of microbiological response within 12 months (based on sputum AFB culture negativity and negative culture conversion) after treatment between 2 groups: - Group treated with conventional regimens - Group treated with conventional regimens PLUS TIG for 2 or 4 weeks during the initial phase 3. Conventional agents used: - AMK, IMP, CFX - MAC, CFZ, LZD, RFB	1. Short-term iv TIG treatment during a 1-month initial phase may ↑ early microbiological response in *M. abscessus* lung disease 2. Short-term use of TIG does not ↑ the long-term culture conversion rate of *M. abscessus* lung disease 3. ↑↑ AFB culture negativity rate at 1 month in the TIG group vs. non-TG group (89% vs. 50%) 4. ↑ culture conversion within 12 months in the non-TIG group vs. TG-group (44% vs. 26%)
Budi, 2023 [100]	OMC	1. Evaluation of murine models using *C. difficile* VPI 10463 2. OMC vs. VAN: * Severe model: - Survival rates - Weight loss - Disease severity - *C. difficile* production * Non-severe model: Addition of Gs 3. Additional assessment: - Colon histology - Bile acid analysis - Spore shedding - 16S sequencing	1. OMC vs. VAN: * Severe model: - Survival rates: 60% vs. 13.3% - ↓ weight loss - ↓ disease severity * Non-severe model: all mice survived with G-antibiotic therapy vs. 60% antibiotics alone 2. ↓ changes in bile acids and microbiota composition in the omadacycline group 3. Germinant–antibiotic combinations showed ↑ outcomes at preventing rCDI vs. antibiotics alone, without spore release or ↑ toxin production at 15 days
Singh, 2024 [94]	OMC	1. PK/PD experiments for the treatment of MDR-TB with OMC 2. Strains that were used: - Mtb H37Rv - MDR-TB strain 16D	1. OMC shows efficacy against both drug-susceptible TB and MDR-TB 2. PK/PD target exposure: AUC0–24/MIC of 26.93 3. MIC breakpoint for the 300 mg daily oral dose >4 mg/L 4. Routine clinical assays for slow-growing bacteria face a disadvantage when testing OMC MICs due to its ↑ degradation rate of 50% in solution at the standard incubation temperature of 37 °C

Abbreviations: AFB, acid-fast bacillus; AMK, amikacin; AUC, area under the curve; CDI, *Clostridioides difficile* infection; CFX, cefoxitin; CFZ, clofazimine; ERV, eravacycline; FDX, fidaxomicin; Gs, germinants; IMP, imipenem; LPS, lipopolysaccharides; LZD, linezolid; MAC, macrolides; MDR-TB, multidrug-resistant *Mycobacterium tuberculosis*; MIC, minimum inhibitory concentration; Mtb, *Mycobacterium tuberculosis*; MTZ, metronidazole; OMC, omadacycline; PD, pharmacodynamics; PK, pharmacokinetics; rCDI, recurrent *Clostridioides difficile* infection; RFB, rifabutin; RT027, *Clostridioides* ribotype 027; TET, tetracycline; TG, tigecycline; VAN, vancomycin; WGC, whole-genome sequencing; ↑, increase; ↓ decrease.

## Abbreviations

ABC: ATP binding cassette; ABSSSI, acute bacterial skin and soft tissue infection; AFB, acid-fast bacillus; Akt, serine/threonine kinase 1; AMK, amikacin; AML, acute myeloid leukemia; AMPK, adenosine monophosphate-activated protein kinase; ARDS, acute respiratory distress syndrome; AUC, area under the curve; BCL-2, B-cell lymphoma 2; CABP, community-acquired bacterial pneumonia; CAC, colitis-associated colorectal cancer; CDI, *Clostridioides difficile* infection; CDK, cyclin-dependent kinase; CFX, cefoxitin; CFZ, clofazimine; cIAI, complicated intra-abdominal infection; CML, chronic myeloid leukemia; CNSAb, carbapenem-non-susceptible; CRAB, carbapenem-resistant *Acinetobacter baumannii*; CRE, carbapenem-resistant Enterobacterales; CRKP, carbapenem-resistant *Klebsiella pneumoniae*; cUTI, complicated urinary tract infections; DOX, doxycycline; ERK1/2, extracellular signal-regulated protein kinases 1 and 2; ERV, eravacycline; ESBL, extended spectrum beta-lactamase; EUCAST, the European Committee on Antimicrobial Susceptibility Testing; FDA, Food and Drug Administration; FDX, fidaxomicin; Gs, germinants; IGNITE, Infections Treated With Eravacycline; IL-, interleukins; IMP, imipenem; INR, international normalized ratio; LPS, lipopolysaccharides; LZD, linezolid; MAB, *Mycobacterium abscessus*; MABC, *Mycobacterium abscessus* complex; MAC, macrolides; MATE, multi-antimicrobial extrusion protein; MBC, minimum bactericidal concentration; MDR, multidrug-resistant; MDR-TB, multidrug-resistant *Mycobacterium tuberculosis;* MIC, minimum inhibitory concentration; MIN, minocycline; MIP, macrophage inflammatory protein; MFS, major facilitator superfamily; MRSA, methicillin-resistant *Staphylococcus aureus*; MRSE, methicillin-resistant *Staphylococcus epidermidis*; Mtb, *Mycobacterium tuberculosis*; mTOR, mammalian target of rapamycin; MTZ, metronidazole; NF-κB, nuclear factor-kappa B; NLRP3, nucleotide-binding domain, leucine-rich-containing family, pyrin domain-containing-3; NO, nitric oxide; NTM, non-tuberculous mycobacteria; OMC, omadacycline; OMP, outer membrane protein; OPTIC, Omadacycline for Pneumonia Treatment In the Community; OxPhos, oxidative phosphorylation; p38, p38 mitogen-activated protein kinase; PD, pharmacodynamics; PDAC, pancreatic ductal adenocarcinoma; PI3K, phosphatidylinositol-3 kinase; PK, pharmacokinetics; PSI, pneumonia severity index; rCDI, recurrent *Clostridioides difficile* infection; RCT, randomized-control trial; RFB, rifabutin; RGM, rapidly growing mycobacteria; RND, resistance nodulation cell division; ROS, reactive oxygen species; RRF, ribosome recycling factor; RT027, *Clostridioides difficile* ribotype 027; SIADH, syndrome of inappropriate antidiuretic hormone secretion; SMR, small multidrug resistance; TET, tetracycline; TG, tigecycline; Th cell, T-helper cell; TKI, tyrosine kinase inhibitor; TMP-SMZ, trimethoprim–sulfamethoxazole; TNF-α, tumor necrosis factor-alpha; UTI, urinary tract infection; VAN, vancomycin; VRE, Vancomycin Resistant Enterococci; WGC, whole-genome sequencing; XDR, extensively drug-resistant.

## References

[1] Nelson M.L., Levy S.B. (2011). The history of the tetracyclines. Ann. N. Y. Acad. Sci..

[2] Linsell W.D., Fletcher A.P. (1950). Laboratory and clinical experience with terramycin hydrochloride. Br. Med. J..

[3] Roberts M.C. (2003). Tetracycline therapy: Update. Clin. Infect. Dis..

[4] Miell J., Dhanjal P., Jamookeeah C. (2015). Evidence for the use of demeclocycline in the treatment of hyponatraemia secondary to SIADH: A systematic review. Int. J. Clin. Pract..

[5] Navarro-Triviño F.J., Pérez-López I., Ruiz-Villaverde R. (2020). Doxycycline, an Antibiotic or an Anti-Inflammatory Agent? The Most Common Uses in Dermatology. Actas Dermosifiliogr. (Engl. Ed.).

[6] Garrido-Mesa N., Zarzuelo A., Gálvez J. (2013). Minocycline: Far beyond an antibiotic. Br. J. Pharmacol..

[7] Singh S., Khanna D., Kalra S. (2021). Minocycline and Doxycycline: More than Antibiotics. Curr. Mol. Pharmacol..

[8] Rassouli A., Shihmani B., Mehrzad J., Shokrpoor S. (2023). The immunomodulatory effect of minocycline on gene expression of inflammation related cytokines in lipopolysaccharide-treated human peripheral blood mononuclear cells. Anim. Biotechnol..

[9] Jung E., Gademann K. (2023). Clinically Approved Antibiotics from 2010 to 2022. Chimia.

[10] Reynolds R.V., Yeung H., Cheng C.E., Cook-Bolden F., Desai S.R., Druby K.M., Freeman E.E., Keri J.E., Stein Gold L.F., Tan J.K.L. (2024). Guidelines of care for the management of acne vulgaris. J. Am. Acad. Dermatol..

[11] Fuoco D. (2012). Classification Framework and Chemical Biology of Tetracycline Structure-Based Drugs. Antibiotics.

[12] Chopra I., Roberts M. (2001). Tetracycline antibiotics: Mode of action, applications, molecular biology, and epidemiology of bacterial resistance. Microbiol. Mol. Biol. Rev..

[13] Barrenechea V., Vargas-Reyes M., Quiliano M., Milón P. (2021). A Complementary Mechanism of Bacterial mRNA Translation Inhibition by Tetracyclines. Front. Microbiol..

[14] LaPlante K.L., Dhand A., Wright K., Lauterio M. (2022). Re-establishing the utility of tetracycline-class antibiotics for current challenges with antibiotic resistance. Ann. Med..

[15] Greer N.D. (2006). Tigecycline (Tygacil): The first in the glycylcycline class of antibiotics. Proc. Bayl. Univ. Med. Cent..

[16] Zhanel G.G., Cheung D., Adam H., Zelenitsky S., Golden A., Schweizer F., Gorityala B., Lagacé-Wiens P.R., Walkty A., Gin A.S. (2016). Review of Eravacycline, a Novel Fluorocycline Antibacterial Agent. Drugs.

[17] Zhanel G.G., Esquivel J., Zelenitsky S., Lawrence C.K., Adam H.J., Golden A., Hink R., Berry L., Schweizer F., Zhanel M.A. (2020). Omadacycline: A Novel Oral and Intravenous Aminomethylcycline Antibiotic Agent. Drugs.

[18] Zhanel G.G., Homenuik K., Nichol K., Noreddin A., Vercaigne L., Embil J., Gin A., Karlowsky J.A., Hoban D.J. (2004). The glycylcyclines: A comparative review with the tetracyclines. Drugs.

[19] Alegun O., Pandeya A., Cui J., Ojo I., Wei Y. (2021). Donnan Potential across the Outer Membrane of Gram-Negative Bacteria and Its Effect on the Permeability of Antibiotics. Antibiotics.

[20] Prajapati J.D., Kleinekathöfer U., Winterhalter M. (2021). How to Enter a Bacterium: Bacterial Porins and the Permeation of Antibiotics. Chem. Rev..

[21] Olson M.W., Ruzin A., Feyfant E., Rush T.S., O’Connell J., Bradford P.A. (2006). Functional, biophysical, and structural bases for antibacterial activity of tigecycline. Antimicrob. Agents Chemother..

[22] Stein G.E., Babinchak T. (2013). Tigecycline: An update. Diagn. Microbiol. Infect. Dis..

[23] Grossman T.H., Starosta A.L., Fyfe C., O’Brien W., Rothstein D.M., Mikolajka A., Wilson D.N., Sutcliffe J.A. (2012). Target- and resistance-based mechanistic studies with TP-434, a novel fluorocycline antibiotic. Antimicrob. Agents Chemother..

[24] Draper M.P., Weir S., Macone A., Donatelli J., Trieber C.A., Tanaka S.K., Levy S.B. (2014). Mechanism of action of the novel aminomethylcycline antibiotic omadacycline. Antimicrob. Agents Chemother..

[25] Meagher A.K., Ambrose P.G., Grasela T.H., Ellis-Grosse E.J. (2005). The pharmacokinetic and pharmacodynamic profile of tigecycline. Clin. Infect. Dis..

[26] Barbour A., Schmidt S., Ma B., Schiefelbein L., Rand K.H., Burkhardt O., Derendorf H. (2009). Clinical pharmacokinetics and pharmacodynamics of tigecycline. Clin. Pharmacokinet..

[27] Zhou C.C., Huang F., Zhang J.M., Zhuang Y.G. (2022). Population Pharmacokinetics of Tigecycline: A Systematic Review. Drug Des. Devel. Ther..

[28] Li M.X., Li N., Zhu L.Q., Liu W. (2020). Optimization of tigecycline dosage regimen for different infections in the patients with hepatic or renal impairment. J. Chemother..

[29] Rafailidis P., Panagopoulos P., Koutserimpas C., Samonis G. (2024). Current Therapeutic Approaches for Multidrug-Resistant and Extensively Drug-Resistant *Acinetobacter baumannii* Infections. Antibiotics.

[30] McCarthy M.W. (2019). Clinical Pharmacokinetics and Pharmacodynamics of Eravacycline. Clin. Pharmacokinet..

[31] Zou X., Jin S., Chen L., Li J., Zhang X., Zhou H., Li X., Huang H. (2023). Antibacterial Activity of Eravacycline Against Carbapenem-Resistant Gram-Negative Isolates in China: An in vitro Study. Infect. Drug Resist..

[32] Scott L.J. (2019). Eravacycline: A Review in Complicated Intra-Abdominal Infections. Drugs.

[33] Rodvold K.A., Pai M.P. (2019). Pharmacokinetics and Pharmacodynamics of Oral and Intravenous Omadacycline. Clin. Infect. Dis..

[34] Rodvold K.A., Burgos R.M., Tan X., Pai M.P. (2020). Omadacycline: A Review of the Clinical Pharmacokinetics and Pharmacodynamics. Clin. Pharmacokinet..

[35] Trang M., Lakota E.A., Safir M.C., Bhavnani S.M., Friedrich L., Steenbergen J.N., McGovern P.C., Tzanis E., Rubino C.M. (2023). Evaluation of the Impact of Comorbidities on Omadacycline Pharmacokinetics. Antimicrob. Agents Chemother..

[36] Cilloniz C., Torres A. (2023). The pharmacokinetic evaluation of omadacycline (Oral Only Dosing Regimen) for the treatment of Community-Acquired Bacterial Pneumonia (CABP). Expert Opin. Drug Metab. Toxicol..

[37] Yaghoubi S., Zekiy A.O., Krutova M., Gholami M., Kouhsari E., Sholeh M., Ghafouri Z., Maleki F. (2022). Tigecycline antibacterial activity, clinical effectiveness, and mechanisms and epidemiology of resistance: Narrative review. Eur. J. Clin. Microbiol. Infect. Dis..

[38] Betriu C., Culebras E., Gómez M., Rodríguez-Avial I., Picazo J.J. (2005). In vitro activity of tigecycline against Bacteroides species. J. Antimicrob. Chemother..

[39] Sorlózano A., Gutiérrez J., Salmerón A., Luna J.D., Martínez-Checa F., Román J., Piédrola G. (2006). Activity of tigecycline against clinical isolates of Staphylococcus aureus and extended-spectrum beta-lactamase-producing Escherichia coli in Granada, Spain. Int. J. Antimicrob. Agents.

[40] Kechagias K.S., Chorepsima S., Triarides N.A., Falagas M.E. (2020). Tigecycline for the treatment of patients with Clostridium difficile infection: An update of the clinical evidence. Eur. J. Clin. Microbiol. Infect. Dis..

[41] Zhang T., Du J., Dong L., Wang F., Zhao L., Jia J., Wang C., Cheng M., Yu X., Huang H. (2023). In Vitro Antimicrobial Activities of Tigecycline, Eravacycline, Omadacycline, and Sarecycline against Rapidly Growing Mycobacteria. Microbiol. Spectr..

[42] Morrissey I., Olesky M., Hawser S., Lob S.H., Karlowsky J.A., Corey G.R., Bassetti M., Fyfe C. (2020). In Vitro Activity of Eravacycline against Gram-Negative Bacilli Isolated in Clinical Laboratories Worldwide from 2013 to 2017. Antimicrob. Agents Chemother..

[43] Zeng W., Zhang X., Liu Y., Zhang Y., Xu M., Wang S., Sun Y., Zhou T., Chen L. (2022). In vitro antimicrobial activity and resistance mechanisms of the new generation tetracycline agents, eravacycline, omadacycline, and tigecycline against clinical Staphylococcus aureus isolates. Front. Microbiol..

[44] Brauncajs M., Bielec F., Macieja A., Pastuszak-Lewandoska D. (2023). In Vitro Activity of Eravacycline against Carbapenemase-Producing Gram-Negative Bacilli Clinical Isolates in Central Poland. Biomedicines.

[45] Alosaimy S., Morrisette T., Lagnf A.M., Rojas L.M., King M.A., Pullinger B.M., Hobbs A.L.V., Perkins N.B., Veve M.P., Bouchard J. (2022). Clinical Outcomes of Eravacycline in Patients Treated Predominately for Carbapenem-Resistant *Acinetobacter baumannii*. Microbiol. Spectr..

[46] Alexander C., Hill D. (2024). A Retrospective Case-Control Study of Eravacycline for the Treatment of Carbapenem-Resistant Acinetobacter Infections in Patients With Burn Injuries. J. Burn Care Res..

[47] Wu J., Zhang G., Zhao Q., Wang L., Yang J., Cui J. (2023). In vitro Antimicrobial Activity and Dose Optimization of Eravacycline and Other Tetracycline Derivatives Against Levofloxacin-Non-Susceptible and/or Trimethoprim-Sulfamethoxazole-Resistant Stenotrophomonas maltophilia. Infect. Drug Resist..

[48] Hawser S., Kothari N., Monti F., Morrissey I., Siegert S., Hodges T. (2023). In vitro activity of eravacycline and comparators against Gram-negative and Gram-positive bacterial isolates collected from patients globally between 2017 and 2020. J. Glob. Antimicrob. Resist..

[49] Karlowsky J.A., Steenbergen J., Zhanel G.G. (2019). Microbiology and Preclinical Review of Omadacycline. Clin. Infect. Dis..

[50] Pfaller M.A., Huband M.D., Shortridge D., Flamm R.K. (2020). Surveillance of Omadacycline Activity Tested against Clinical Isolates from the United States and Europe: Report from the SENTRY Antimicrobial Surveillance Program, 2016 to 2018. Antimicrob. Agents Chemother..

[51] Abbey T., Vialichka A., Jurkovic M., Biagi M., Wenzler E. (2022). Activity of Omadacycline Alone and in Combination against Carbapenem-Nonsusceptible *Acinetobacter baumannii* with Varying Minocycline Susceptibility. Microbiol. Spectr..

[52] Deolankar M.S., Carr R.A., Fliorent R., Roh S., Fraimow H., Carabetta V.J. (2022). Evaluating the Efficacy of Eravacycline and Omadacycline against Extensively Drug-Resistant *Acinetobacter baumannii* Patient Isolates. Antibiotics.

[53] O’Donnell J.N., Putra V., Maring B.L., Ozer E.A., Belfiore G.M., Rhodes N.J. (2022). Effect of omadacycline alone and in combination with meropenem against carbapenem-resistant *Acinetobacter baumannii* isolates. J. Glob. Antimicrob. Resist..

[54] Grossman T.H. (2016). Tetracycline Antibiotics and Resistance. Cold Spring Harb. Perspect. Med..

[55] Vanbaelen T., Manoharan-Basil S.S., Kenyon C. (2024). 45 years of tetracycline post exposure prophylaxis for STIs and the risk of tetracycline resistance: A systematic review and meta-analysis. BMC Infect. Dis..

[56] Jensen J.S., Unemo M. (2024). Antimicrobial treatment and resistance in sexually transmitted bacterial infections. Nat. Rev. Microbiol..

[57] Sun C., Yu Y., Hua X. (2023). Resistance mechanisms of tigecycline in *Acinetobacter baumannii*. Front. Cell. Infect. Microbiol..

[58] Roch M., Sierra R., Andrey D.O. (2023). Antibiotic heteroresistance in ESKAPE pathogens, from bench to bedside. Clin. Microbiol. Infect..

[59] Khlaif M.M., Hussein N.H. (2022). Sequencing analysis of tigecycline resistance among non-susceptible in three species of G-ve bacteria isolated from clinical specimens in Baghdad. Mol. Biol. Rep..

[60] Sun X., Zhang B., Xu G., Chen J., Shang Y., Lin Z., Yu Z., Zheng J., Bai B. (2021). In Vitro Activity of the Novel Tetracyclines, Tigecycline, Eravacycline, and Omadacycline, Against Moraxella catarrhalis. Ann. Lab. Med..

[61] Okada U., Murakami S. (2022). Structural and functional characteristics of the tripartite ABC transporter. Microbiology.

[62] Dong N., Zeng Y., Wang Y., Liu C., Lu J., Cai C., Liu X., Chen Y., Wu Y., Fang Y. (2022). Distribution and spread of the mobilized RND efflux pump gene cluster tmexCD-toprJ in clinical Gram-negative bacteria: A molecular epidemiological study. Lancet Microbe.

[63] Burata O.E., Yeh T.J., Macdonald C.B., Stockbridge R.B. (2022). Still rocking in the structural era: A molecular overview of the small multidrug resistance (SMR) transporter family. J. Biol. Chem..

[64] Shi Y., Hua X., Xu Q., Yang Y., Zhang L., He J., Mu X., Hu L., Leptihn S., Yu Y. (2020). Mechanism of eravacycline resistance in *Acinetobacter baumannii* mediated by a deletion mutation in the sensor kinase adeS, leading to elevated expression of the efflux pump AdeABC. Infect. Genet. Evol..

[65] Anyanwu M.U., Nwobi O.C., Okpala C.O.R., Ezeonu I.M. (2022). Mobile Tigecycline Resistance: An Emerging Health Catastrophe Requiring Urgent One Health Global Intervention. Front. Microbiol..

[66] Sun J., Chen C., Cui C.Y., Zhang Y., Liu X., Cui Z.H., Ma X.Y., Feng Y., Fang L.X., Lian X.L. (2019). Plasmid-encoded tet(X) genes that confer high-level tigecycline resistance in Escherichia coli. Nat. Microbiol..

[67] Li R., Jiang Y., Peng K., Wang Y., Wang M., Liu Y., Wang Z. (2022). Phenotypic and genomic analysis reveals Riemerella anatipestifer as the potential reservoir of tet(X) variants. J. Antimicrob. Chemother..

[68] Hsieh Y.C., Wu J.W., Chen Y.Y., Quyen T.L.T., Liao W.C., Li S.W., Chen Y.C., Pan Y.J. (2021). An Outbreak of tet(X6)-Carrying Tigecycline-Resistant *Acinetobacter baumannii* Isolates with a New Capsular Type at a Hospital in Taiwan. Antibiotics.

[69] Wang Q., Lei C., Cheng H., Yang X., Huang Z., Chen X., Ju Z., Zhang H., Wang H. (2022). Widespread Dissemination of Plasmid-Mediated Tigecycline Resistance Gene tet(X4) in Enterobacterales of Porcine Origin. Microbiol. Spectr..

[70] Chirabhundhu N., Luk-In S., Phuadraksa T., Wichit S., Chatsuwan T., Wannigama D.L., Yainoy S. (2024). Occurrence and mechanisms of tigecycline resistance in carbapenem- and colistin-resistant Klebsiella pneumoniae in Thailand. Sci. Rep..

[71] Alosaimy S., Abdul-Mutakabbir J.C., Kebriaei R., Jorgensen S.C.J., Rybak M.J. (2020). Evaluation of Eravacycline: A Novel Fluorocycline. Pharmacotherapy.

[72] Hua X., He J., Wang J., Zhang L., Zhang L., Xu Q., Shi K., Leptihn S., Shi Y., Fu X. (2021). Novel tigecycline resistance mechanisms in *Acinetobacter baumannii* mediated by mutations in adeS, rpoB and rrf. Emerg. Microbes Infect..

[73] Heidrich C.G., Mitova S., Schedlbauer A., Connell S.R., Fucini P., Steenbergen J.N., Berens C. (2016). The Novel Aminomethylcycline Omadacycline Has High Specificity for the Primary Tetracycline-Binding Site on the Bacterial Ribosome. Antibiotics.

[74] Shi J., Cheng J., Liu S., Zhu Y., Zhu M. (2024). *Acinetobacter baumannii*: An evolving and cunning opponent. Front. Microbiol..

[75] Park S., Kim H., Ko K.S. (2023). Reduced virulence in tigecycline-resistant Klebsiella pneumoniae caused by overexpression of ompR and down-regulation of ompK35. J. Biomed. Sci..

[76] Xu C., Wei X., Jin Y., Bai F., Cheng Z., Chen S., Pan X., Wu W. (2022). Development of Resistance to Eravacycline by Klebsiella pneumoniae and Collateral Sensitivity-Guided Design of Combination Therapies. Microbiol. Spectr..

[77] Ajiboye T.O., Skiebe E., Wilharm G. (2018). Contributions of RecA and RecBCD DNA repair pathways to the oxidative stress response and sensitivity of *Acinetobacter baumannii* to antibiotics. Int. J. Antimicrob. Agents.

[78] Babinchak T., Ellis-Grosse E., Dartois N., Rose G.M., Loh E., Tigecycline 301 Study Group, Tigecycline 306 Study Group (2005). The efficacy and safety of tigecycline for the treatment of complicated intra-abdominal infections: Analysis of pooled clinical trial data. Clin. Infect. Dis..

[79] Ellis-Grosse E.J., Babinchak T., Dartois N., Rose G., Loh E., Tigecycline 300 cSSSI Study Group, Tigecycline 305 cSSSI Study Group (2005). The efficacy and safety of tigecycline in the treatment of skin and skin-structure infections: Results of 2 double-blind phase 3 comparison studies with vancomycin-aztreonam. Clin. Infect. Dis..

[80] Tanaseanu C., Bergallo C., Teglia O., Jasovich A., Oliva M.E., Dukart G., Dartois N., Cooper C.A., Gandjini H., Mallick R. (2008). Integrated results of 2 phase 3 studies comparing tigecycline and levofloxacin in community-acquired pneumonia. Diagn. Microbiol. Infect. Dis..

[81] Solomkin J., Evans D., Slepavicius A., Lee P., Marsh A., Tsai L., Sutcliffe J.A., Horn P. (2017). Assessing the Efficacy and Safety of Eravacycline vs Ertapenem in Complicated Intra-abdominal Infections in the Investigating Gram-Negative Infections Treated With Eravacycline (IGNITE 1) Trial: A Randomized Clinical Trial. JAMA Surg..

[82] Solomkin J.S., Gardovskis J., Lawrence K., Montravers P., Sway A., Evans D., Tsai L. (2019). IGNITE4: Results of a Phase 3, Randomized, Multicenter, Prospective Trial of Eravacycline vs Meropenem in the Treatment of Complicated Intraabdominal Infections. Clin. Infect. Dis..

[83] Meng R., Guan X., Sun L., Fei Z., Li Y., Luo M., Ma A., Li H. (2022). The efficacy and safety of eravacycline compared with current clinically common antibiotics in the treatment of adults with complicated intra-abdominal infections: A Bayesian network meta-analysis. Front. Med..

[84] Lan S.H., Chang S.P., Lai C.C., Lu L.C., Chao C.M. (2019). The Efficacy and Safety of Eravacycline in the Treatment of Complicated Intra-Abdominal Infections: A Systemic Review and Meta-Analysis of Randomized Controlled Trials. J. Clin. Med..

[85] Rodriguez G.D., Warren N., Yashayev R., Chitra S., Amodio-Groton M., Wright K. (2023). Omadacycline in the treatment of community-acquired bacterial pneumonia in patients with comorbidities: A post-hoc analysis of the phase 3 OPTIC trial. Front. Med..

[86] Esposito S., Bassetti M., Concia E., De Simone G., De Rosa F.G., Grossi P., Novelli A., Menichetti F., Petrosillo N., Tinelli M. (2017). Italian Society of Infectious and Tropical Diseases. Diagnosis and management of skin and soft-tissue infections (SSTI). A literature review and consensus statement: An update. J. Chemother..

[87] Alsaad N., Wilffert B., van Altena R., de Lange W.C., van der Werf T.S., Kosterink J.G., Alffenaar J.W. (2014). Potential antimicrobial agents for the treatment of multidrug-resistant tuberculosis. Eur. Respir. J..

[88] Daley C.L., Iaccarino J.M., Lange C., Cambau E., Wallace R.J., Andrejak C., Böttger E.C., Brozek J., Griffith D.E., Guglielmetti L. (2020). Treatment of nontuberculous mycobacterial pulmonary disease: An official ATS/ERS/ESCMID/IDSA clinical practice guideline. Eur. Respir. J..

[89] Kwon Y.S., Levin A., Kasperbauer S.H., Huitt G.A., Daley C.L. (2019). Efficacy and safety of tigecycline for Mycobacterium abscessus disease. Respir. Med..

[90] Kaushik A., Ammerman N.C., Martins O., Parrish N.M., Nuermberger E.L. (2019). In Vitro Activity of New Tetracycline Analogs Omadacycline and Eravacycline against Drug-Resistant Clinical Isolates of Mycobacterium abscessus. Antimicrob. Agents Chemother..

[91] Brown-Elliott B.A., Wallace R.J. (2022). In Vitro Susceptibility Testing of Eravacycline against Nontuberculous Mycobacteria. Antimicrob. Agents Chemother..

[92] Rizzo A.R., Moniri N.H. (2022). Omadacycline for management of Mycobacterium abscessus infections: A review of its effectiveness, place in therapy, and considerations for use. BMC Infect. Dis..

[93] El Ghali A., Morrisette T., Alosaimy S., Lucas K., Tupayachi-Ortiz M.G., Vemula R., Wadle C., Philley J.V., Mejia-Chew C., Hamad Y. (2023). Long-term evaluation of clinical success and safety of omadacycline in nontuberculous mycobacteria infections: A retrospective, multicenter cohort of real-world health outcomes. Antimicrob. Agents Chemother..

[94] Singh S., Gumbo T., Boorgula G.D., Thomas T.A., Philley J.V., Srivastava S. (2024). Omadacycline pharmacokinetics/pharmacodynamics and efficacy against multidrug-resistant Mycobacterium tuberculosis in the hollow fiber system model. Antimicrob. Agents Chemother..

[95] Jump R.L., Li Y., Pultz M.J., Kypriotakis G., Donskey C.J. (2011). Tigecycline exhibits inhibitory activity against Clostridium difficile in the colon of mice and does not promote growth or toxin production. Antimicrob. Agents Chemother..

[96] Phillips E.C., Warren C.A., Ma J.Z., Madden G.R. (2022). Impact of Tigecycline on C. difficile Outcomes: Case Series and Propensity-Matched Retrospective Study. Antimicrob. Agents Chemother..

[97] Buckley A.M., Altringham J., Clark E., Bently K., Spittal W., Ewin D., Wilkinson V., Davis G., Moura I.B., Wilcox M.H. (2021). Eravacycline, a novel tetracycline derivative, does not induce Clostridioides difficile infection in an in vitro human gut model. J. Antimicrob. Chemother..

[98] Bassères E., Begum K., Lancaster C., Gonzales-Luna A.J., Carlson T.J., Miranda J., Rashid T., Alam M.J., Eyre D.W., Wilcox M.H. (2020). In vitro activity of eravacycline against common ribotypes of Clostridioides difficile. J. Antimicrob. Chemother..

[99] Skinner A.M., Petrella L.A., Cheknis A., Johnson S. (2023). Antimicrobial susceptibility of Clostridioides difficile to omadacycline and comparator antimicrobials. J. Antimicrob. Chemother..

[100] Budi N.D., Godfrey J.J., Safdar N., Shukla S.K., Rose W.E. (2023). Efficacy of Omadacycline or Vancomycin Combined With Germinants for Preventing Clostridioides difficile Relapse in a Murine Model. J. Infect. Dis..

[101] Jo J., Hu C., Begum K., Wang W., Le T.M., Agyapong S., Hanson B.M., Ayele H., Lancaster C., Jahangir Alam M. (2024). Fecal Pharmacokinetics and Gut Microbiome Effects of Oral Omadacycline Versus Vancomycin in Healthy Volunteers. J. Infect. Dis..

[102] Yang J.C., Lee P.I., Hsueh P.R. (2010). In vitro activity of nemonoxacin, tigecycline, and other antimicrobial agents against Helicobacter pylori isolates in Taiwan, 1998–2007. Eur. J. Clin. Microbiol. Infect. Dis..

[103] Monno R., Fumarola L., Capolongo C., Calia C., Pazzani C., Ierardi E., Miragliotta G. (2015). Susceptibility of Helicobacter pylori to Antibiotics Including Tigecycline. J. Med. Microbiol. Diagn..

[104] Yang Y., Bian L., Hang X., Yan C., Huang Y., Ye F., Zhang G., Jin G., Bi H. (2020). In vitro activity of new tetracycline analogues omadacycline and eravacycline against clinical isolates of Helicobacter pylori collected in China. Diagn. Microbiol. Infect. Dis..

[105] Wu G., Abraham T., Saad N. (2014). Role of Tigecycline for the Treatment of Urinary Tract Infections. J. Pharm. Technol..

[106] Liu Y.X., Le K.J., Shi H.Y., Zhang Z.L., Cui M., Zhong H., Yu Y.T., Gu Z.C. (2021). Efficacy and safety of tigecycline for complicated urinary tract infection: A systematic review. Transl. Androl. Urol..

[107] Charles R., Adhikari S.D., Mittal A., Chaudhuri S., Gupta M., Khot W., Schito M., Gupta N. (2022). Role of tigecycline in the treatment of urinary tract infections: A systematic review of published case reports. InfezMed.

[108] Stone T.J., Kilic A., Williamson J.C., Palavecino E.L. (2023). In Vitro Activity of Omadacycline and Comparator Antibiotics against Extended-Spectrum Beta-Lactamase-Producing Escherichia coli and Klebsiella pneumoniae Urinary Isolates. Antibiotics.

[109] Kim S.R., Jang M., Kim S.Y., Kim D.H., Jhun B.W. (2022). Outcomes of Short-Term Tigecycline-Containing Regimens for Mycobacterium abscessus Pulmonary Disease. Antimicrob. Agents Chemother..

[110] Karau M.J., Schmidt-Malan S.M., Cunningham S.A., Mandrekar J.N., Pritt B.S., Keepers T.R., Serio A.W., Chitra S., Patel R. (2022). Activity of Omadacycline in Rat Methicillin-Resistant Staphylococcus aureus Osteomyelitis. Antimicrob. Agents Chemother..

[111] Morrisette T., Stamper K.C., Lev K.L., Kebriaei R., Holger D.J., Abdul-Mutakabbir J.C., Kunz Coyne A.J., Rybak M.J. (2023). Evaluation of Omadacycline Alone and in Combination with Rifampin against Staphylococcus aureus and Staphylococcus epidermidis in an In Vitro Pharmacokinetic/Pharmacodynamic Biofilm Model. Antimicrob. Agents Chemother..

[112] Ni W., Yang D., Guan J., Xi W., Zhou D., Zhao L., Cui J., Xu Y., Gao Z., Liu Y. (2021). In vitro and in vivo synergistic effects of tigecycline combined with aminoglycosides on carbapenem-resistant Klebsiella pneumoniae. J. Antimicrob. Chemother..

[113] Nulsopapon P., Nasomsong W., Pongchaidecha M., Changpradub D., Juntanawiwat P., Santimaleeworagun W. (2021). The Synergistic Activity and Optimizing Doses of Tigecycline in Combination with Aminoglycosides against Clinical Carbapenem-Resistant Klebsiella pneumoniae Isolates. Antibiotics.

[114] Zavascki A.P., Klee B.O., Bulitta J.B. (2017). Aminoglycosides against carbapenem-resistant Enterobacteriaceae in the critically ill: The pitfalls of aminoglycoside susceptibility. Expert Rev. Anti. Infect. Ther..

[115] Yang F., Chen P., Wang H., Xing X., Wang S., Ishaq H.M., Liao W. (2022). Comparative Minimal Inhibitory and Mutant Prevention Concentration of Eight Antimicrobial Agents Against Klebsiella pneumoniae. Microb. Drug Resist..

[116] Rahul R., Maheswary D., Damodaran N., Leela K.V. (2023). Eravacycline -Synergistic activity with other antimicrobials in carbapenem resistant isolates of Escherichia coli and *Acinetobacter baumannii*. Diagn. Microbiol. Infect. Dis..

[117] Li J., Fu Y., Zhang J., Wang Y., Zhao Y., Fan X., Yu L., Wang Y., Zhang X., Li C. (2019). Efficacy of tigecycline monotherapy versus combination therapy with other antimicrobials against carbapenem-resistant *Acinetobacter baumannii* sequence type 2 in Heilongjiang Province. Ann. Palliat. Med..

[118] Ozger H.S., Cuhadar T., Yildiz S.S., Demirbas Gulmez Z., Dizbay M., Guzel Tunccan O., Kalkanci A., Simsek H., Unaldi O. (2019). In vitro activity of eravacycline in combination with colistin against carbapenem-resistant A. baumannii isolates. J. Antibiot..

[119] Li Y., Cui L., Xue F., Wang Q., Zheng B. (2022). Synergism of eravacycline combined with other antimicrobial agents against carbapenem-resistant Enterobacteriaceae and *Acinetobacter baumannii*. J. Glob. Antimicrob. Resist..

[120] Bich Hanh B.T., Quang N.T., Park Y., Heo B.E., Jeon S., Park J.W., Jang J. (2021). Omadacycline Potentiates Clarithromycin Activity Against Mycobacterium abscessus. Front. Pharmacol..

[121] Fujiwara K., Aono A., Asami T., Morimoto K., Kamada K., Morishige Y., Igarashi Y., Chikamatsu K., Murase Y., Yamada H. (2023). In Vitro Synergistic Effects of Omadacycline with Other Antimicrobial Agents against Mycobacterium abscessus. Antimicrob. Agents Chemother..

[122] Rimal B., Nicklas D.A., Panthi C.M., Lippincott C.K., Belz D.C., Ignatius E.H., Deck D.H., Serio A.W., Lamichhane G. (2023). Efficacy of Omadacycline-Containing Regimen in a Mouse Model of Pulmonary Mycobacteroides abscessus Disease. mSphere.

[123] Aziz D.B., Teo J.W.P., Dartois V., Dick T. (2018). Teicoplanin—Tigecycline Combination Shows Synergy Against Mycobacterium abscessus. Front. Microbiol..

[124] Chew K.L., Octavia S., Yeoh S.F., Teo J.W.P. (2021). In Vitro Synergy Testing of Eravacycline in Combination with Clarithromycin and Rifabutin against Mycobacterium abscessus Complex. Microbiol. Spectr..

[125] Ku T.S., Palanisamy S.K., Lee S.A. (2010). Susceptibility of Candida albicans biofilms to azithromycin, tigecycline and vancomycin and the interaction between tigecycline and antifungals. Int. J. Antimicrob. Agents.

[126] Hooper R.W., Ashcraft D.S., Pankey G.A. (2019). In vitro synergy with fluconazole plus doxycycline or tigecycline against clinical Candida glabrata isolates. Med. Mycol..

[127] Li X., Yang H., Duan X., Cui M., Xing W., Zheng S. (2023). Synergistic effect of eravacycline combined with fluconazole against resistant Candida albicans in vitro and in vivo. Expert Rev. Anti. Infect. Ther..

[128] Sun J., Shigemi H., Tanaka Y., Yamauchi T., Ueda T., Iwasaki H. (2015). Tetracyclines downregulate the production of LPS-induced cytokines and chemokines in THP-1 cells via ERK, p38, and nuclear factor-κB signaling pathways. Biochem. Biophys. Rep..

[129] Peukert K., Fox M., Schulz S., Feuerborn C., Frede S., Putensen C., Wrigge H., Kümmerer B.M., David S., Seeliger B. (2021). Inhibition of Caspase-1 with Tetracycline Ameliorates Acute Lung Injury. Am. J. Respir. Crit. Care Med..

[130] Dikshit N., Kale S.D., Khameneh H.J., Balamuralidhar V., Tang C.Y., Kumar P., Lim T.P., Tan T.T., Kwa A.L., Mortellaro A. (2018). NLRP3 inflammasome pathway has a critical role in the host immunity against clinically relevant *Acinetobacter baumannii* pulmonary infection. Mucosal. Immunol..

[131] Yagnik R.M., Benzeroual K.E. (2013). Tigecycline prevents LPS-induced release of pro-inflammatory and apoptotic mediators in neuronal cells. Toxicol. In Vitro.

[132] Cockeran R., Mutepe N.D., Theron A.J., Tintinger G.R., Steel H.C., Stivaktas P.I., Richards G.A., Feldman C., Anderson R. (2012). Calcium-dependent potentiation of the pro-inflammatory functions of human neutrophils by tigecycline in vitro. J. Antimicrob. Chemother..

[133] Elhayek S.Y., Fararjeh M.A., Assaf A.M., Abu-Rish E.Y., Bustanji Y. (2018). Immunomodulatory Effects of Tigecycline in Balb/C Mice. Acta Pharm..

[134] Bryant A.E., Stevens D.L. (2022). Investigating the immunomodulatory activities of omadacycline. J. Antimicrob. Chemother..

[135] Skrtić M., Sriskanthadevan S., Jhas B., Gebbia M., Wang X., Wang Z., Hurren R., Jitkova Y., Gronda M., Maclean N. (2011). Inhibition of mitochondrial translation as a therapeutic strategy for human acute myeloid leukemia. Cancer Cell.

[136] Dong Z., Abbas M.N., Kausar S., Yang J., Li L., Tan L., Cui H. (2019). Biological Functions and Molecular Mechanisms of Antibiotic Tigecycline in the Treatment of Cancers. Int. J. Mol. Sci..

[137] Xu Z., Yan Y., Li Z., Qian L., Gong Z. (2016). The Antibiotic Drug Tigecycline: A Focus on its Promising Anticancer Properties. Front. Pharmacol..

[138] Ruiz-Malagón A.J., Hidalgo-García L., Rodríguez-Sojo M.J., Molina-Tijeras J.A., García F., Diez-Echave P., Vezza T., Becerra P., Marchal J.A., Redondo-Cerezo E. (2023). Tigecycline reduces tumorigenesis in colorectal cancer via inhibition of cell proliferation and modulation of immune response. Biomed. Pharmacother..

[139] Lu Z., Xu N., He B., Pan C., Lan Y., Zhou H., Liu X. (2017). Inhibition of autophagy enhances the selective anti-cancer activity of tigecycline to overcome drug resistance in the treatment of chronic myeloid leukemia. J. Exp. Clin. Cancer Res..

[140] Tan J., Song M., Zhou M., Hu Y. (2017). Antibiotic tigecycline enhances cisplatin activity against human hepatocellular carcinoma through inducing mitochondrial dysfunction and oxidative damage. Biochem. Biophys. Res. Commun..

[141] Hu B., Guo Y. (2019). Inhibition of mitochondrial translation as a therapeutic strategy for human ovarian cancer to overcome chemoresistance. Biochem. Biophys. Res. Commun..

[142] Yang R., Yi L., Dong Z., Ouyang Q., Zhou J., Pang Y., Wu Y., Xu L., Cui H. (2016). Tigecycline inhibits glioma growth by regulating miRNA-199b-5p-HES1-AKT pathway. Mol. Cancer Ther..

[143] Zhong X., Zhao E., Tang C., Zhang W., Tan J., Dong Z., Ding H.F., Cui H. (2016). Antibiotic drug tigecycline reduces neuroblastoma cells proliferation by inhibiting Akt activation in vitro and in vivo. Tumour. Biol..

[144] Xiong Y., Liu W., Huang Q., Wang J., Wang Y., Li H., Fu X. (2018). Tigecycline as a dual inhibitor of retinoblastoma and angiogenesis via inducing mitochondrial dysfunctions and oxidative damage. Sci. Rep..

[145] Jabarin A., Shtar G., Feinshtein V., Mazuz E., Shapira B., Ben-Shabat S., Rokach L. (2024). Eravacycline, an antibacterial drug, repurposed for pancreatic cancer therapy: Insights from a molecular-based deep learning model. Brief. Bioinform..

[146] Rolston K., Gerges B., Nesher L., Shelburne S.A., Prince R., Raad I. (2023). In vitro activity of eravacycline and comparator agents against bacterial pathogens isolated from patients with cancer. JAC Antimicrob. Resist..

[147] Tao R.E., Prajapati S., Pixley J.N., Grada A., Feldman S.R. (2023). Oral Tetracycline-Class Drugs in Dermatology: Impact of Food Intake on Absorption and Efficacy. Antibiotics.

[148] Opal S., File T.M., van der Poll T., Tzanis E., Chitra S., McGovern P.C. (2019). An Integrated Safety Summary of Omadacycline, a Novel Aminomethylcycline Antibiotic. Clin. Infect. Dis..

[149] Wang P.F., Zou H., Zhu J.H., Shi F.E. (2021). Acute pancreatitis caused by tigecycline: A case report and literature review. Medicine.

[150] Eljaaly K., Alghamdi H., Almehmadi H., Aljawi F., Hassan A., Thabit A.K. (2023). Long-term gastrointestinal adverse effects of doxycycline. J. Infect. Dev. Ctries..

[151] Sánchez A.R., Rogers R.S., Sheridan P.J. (2004). Tetracycline and other tetracycline-derivative staining of the teeth and oral cavity. Int. J. Dermatol..

[152] Rusu A., Buta E.L. (2021). The Development of Third-Generation Tetracycline Antibiotics and New Perspectives. Pharmaceutics.

[153] Lan S.H., Chang S.P., Lai C.C., Lu L.C., Chao C.M. (2019). The efficacy and safety of omadacycline in treatment of acute bacterial infection: A systemic review and meta-analysis of randomized controlled trials. Medicine.

[154] Tanaka S.K., Villano S. (2016). In Vitro and In Vivo Assessments of Cardiovascular Effects with Omadacycline. Antimicrob. Agents Chemother..

[155] Kadoyama K., Sakaeda T., Tamon A., Okuno Y. (2012). Adverse event profile of tigecycline: Data mining of the public version of the U.S. Food and Drug Administration adverse event reporting system. Biol. Pharm. Bull..

[156] Zhu Y., Zhao F., Jin P. (2023). Clinical Manifestations and Risk Factors of Tigecycline-Associated Thrombocytopenia. Infect. Drug Resist..

[157] Durães F., Sousa E. (2019). Omadacycline: A Newly Approved Antibacterial from the Class of Tetracyclines. Pharmaceuticals.

[158] Leng B., Xue Y.C., Zhang W., Gao T.T., Yan G.Q., Tang H. (2019). A Retrospective Analysis of the Effect of Tigecycline on Coagulation Function. Chem. Pharm. Bull..

[159] Ray A., Sharma S., Atal S., Sadasivam B., Jhaj R. (2020). Tigecycline-Induced Severe Hypoglycemia in a Non-Diabetic Individual: A Case Report and Brief Review of Tigecycline-Induced Severe Hypoglycemia. Am. J. Case Rep..

[160] Hakeam H.A., Sarkhi K.A., Iansavichene A. (2024). Tigecycline and Hypoglycemia, When and How?. J. Pharm. Technol..

[161] Prasad P., Sun J., Danner R.L., Natanson C. (2012). Excess deaths associated with tigecycline after approval based on noninferiority trials. Clin. Infect. Dis..

[162] McGovern P.C., Wible M., El-Tahtawy A., Biswas P., Meyer R.D. (2013). All-cause mortality imbalance in the tigecycline phase 3 and 4 clinical trials. Int. J. Antimicrob. Agents.

